# Artificial Intelligence for Multi-omics Breast Cancer Subtype Prediction and Discovery: A Systematic Review

**DOI:** 10.34133/csbj.0243

**Published:** 2026-10-26

**Authors:** Deepali Pilodiya, Azadeh Noori Hoshyar, Cameron Foale, Madhu Chetty, Piyushkumar A. Mundra

**Affiliations:** ^1^ Institute of Innovation, Science and Sustainability, Federation University Australia, Ballarat, VIC 3353, Australia.; ^2^ School of Computer, Data and Mathematical Sciences, Western Sydney University, Sydney, NSW 2751, Australia.; ^3^ Children’s Cancer Institute, Health Translational Hub, Sydney, NSW 20, Australia.; ^4^ School of Clinical Medicine, UNSW Medicine & Health, Sydney, NSW 2031, Australia.

## Abstract

Artificial intelligence models integrate molecular omics to reproduce breast cancer subtype labels or discover patient groups, but heterogeneous endpoints, cohorts, and validation hinder interpretation. We searched PubMed/MEDLINE, Europe PMC, and OpenAlex through 2026 September 8, supplemented by citation searching and targeted retrieval through Google Scholar and IEEE Xplore. Eligible studies combined at least 2 molecular omics in one machine-learning model for breast cancer subtype prediction or discovery. Findings were synthesized by endpoint without meta-analysis. Of 705 exported records, 301 duplicates were removed, 404 unique records were screened, and 70 studies were included. Fifty-seven addressed intrinsic subtype or Prediction Analysis of Microarray 50 (PAM50) prediction, and 11, subtype discovery or recovery. Sixty-two publications used The Cancer Genome Atlas Breast Invasive Carcinoma, and 9 used the Molecular Taxonomy of Breast Cancer International Consortium, demonstrating extensive reuse of limited patient populations. Among 59 supervised prediction studies, only 1 independently evaluated a complete multi-omics model in another cohort, and none reported prospective or nested full-pipeline validation. Calibration, uncertainty reporting, and external validation were uncommon, although 34 studies provided code or software. Among 11 discovery studies, 4 assessed resampling stability and 3 attempted independent subtype recovery. All 57 PAM50 studies used expression, and none reported excluding PAM50 classifier genes or close proxies. Thirty-nine included a matched expression-only baseline, but only one tested the complete model independently. Risk of bias was high in 48/59 prediction studies. Graph, hypergraph, attention, and uncertainty-aware models are increasingly used, but evidence of transportability and calibrated clinical value remains limited.

## Introduction

Breast cancer comprises biologically and clinically distinct diseases rather than one homogeneous entity. Gene-expression studies established intrinsic groups commonly described as luminal A, luminal B, human epidermal growth factor receptor 2 (HER2)-enriched, basal-like, and normal-like, and the Prediction Analysis of Microarray 50 (PAM50) assay provided a supervised predictor for assigning those groups [1,2]. Large genomic and transcriptomic studies subsequently showed that substantial heterogeneity also exists within expression-defined classes and that genomic drivers, copy-number states, and immune or stromal features can refine this structure [3]. Clinical practice uses related but nonidentical definitions based on estrogen receptor (ER), progesterone receptor, HER2, and triple-negative status. Consequently, predicting a 5-class PAM50 label, distinguishing hormone-receptor groups, classifying triple-negative breast cancer (TNBC), and discovering *de novo* patient clusters are different tasks with different reference standards and intended uses.

Multi-omics analysis attempts to combine complementary measurements from the same tumor. Messenger RNA (mRNA) reflects the transcriptional state, microRNA (miRNA) captures posttranscriptional regulation, DNA methylation encodes an epigenetic layer, copy-number alteration and somatic mutation describe genomic change, and protein or reverse-phase protein array (RPPA) measurements are closer to pathway activity. Integration is attractive because no single assay fully describes a tumor. It is also difficult: the number of molecular variables is often orders of magnitude larger than the number of patients, complete cases decrease rapidly as modalities are added, batch and platform effects differ across omics, and subtype prevalence is markedly imbalanced. Every apparently technical choice—imputation, normalization, feature selection, graph construction, representation learning, and hyperparameter tuning—can therefore alter the estimated performance.

The literature is also vulnerable to a special form of target dependence. PAM50 labels are generated from the expression of a predefined gene set. A model that receives genome-wide expression and predicts PAM50 can partly reconstruct the assay used to create its target. Such work can be valuable for computational assay reproduction and for studying complementary signals from nontranscriptomic layers, but it is not equivalent to predicting an independently adjudicated clinical phenotype. The distinction matters when interpreting high internal accuracy.

Several reviews provide useful foundations. Picard *et al.* [4] organized general integration strategies, Li *et al.* [5] examined knowledge-guided methods, Ballard *et al.* [6] surveyed deep-learning integration across diseases and downstream tasks, and Shah *et al.* [7] focused on artificial intelligence (AI)-driven breast cancer molecular subtype classification. Table 1 shows how their scopes differ. A remaining need is a reproducible breast-specific evidence audit that identifies every included study, separates endpoint strata, explains what the algorithms actually did, and evaluates the credibility of model validation instead of ranking heterogeneous point estimates.

**Table 1.** Scope and contribution of closely related reviews

Review	Main scope	Primary contribution	Gap addressed here
Picard *et al.* [4]	Multidisease, general integration	Clear early/intermediate/late strategy overview	Breast-specific endpoints, study-level evidence table, and validation audit
Li *et al.* [5]	Knowledge-guided multi-omics learning	Biological priors and knowledge incorporation	Study-level breast cancer evidence and transportability
Ballard *et al.* [6]	Deep learning across diseases/tasks	Architecture-focused synthesis	Endpoint stratification, leakage, calibration, and external validation
Shah *et al.* [7]	Breast cancer molecular subtype AI	Broad catalog of recent models	Auditable searches, detailed evidence tables, orthogonal taxonomy, and formal appraisal
This review	Breast cancer subtype prediction and discovery	Seventy studies; detailed contributions; endpoint-stratified SWiM synthesis	Links architectural development to validation and clinical-evidence limitations

AI, artificial intelligence; SWiM, synthesis without meta-analysis

The review addresses 4 questions:

• Which breast cancer subtype endpoints and cohorts have been studied.
• What each included study contributed and how multi-omics information was fused.
• How representation, interaction, prediction, and interpretation mechanisms evolved.
• Whether validation, leakage prevention, calibration, uncertainty, and reproducibility support transportability. Published point estimates were not used to rank models because a valid ranking would require common patients, labels, splits, preprocessing, and metrics.

## Materials and Methods

### Design, reporting guidance, and protocol

The review followed Preferred Reporting Items for Systematic reviews and Meta-Analyses (PRISMA) 2020 and PRISMA-S [8,9], and the narrative synthesis followed synthesis without meta-analysis (SWiM) [10]. The protocol was not prospectively registered. A written methods record was maintained during the review to document the eligibility criteria, information sources, screening and extraction procedures, synthesis approach, and appraisal framework. It also documented the use of endpoint-applicable denominators, the separation of risk of bias from other evidence characteristics, the PAM50 target-dependence audit, and the patient-population map. These procedures and their operational definitions are described below and in the Supplementary Materials. All counts in the flow diagram are based on saved exports and deduplicated records rather than search-platform hit estimates.

### Eligibility criteria

Eligible reports were English-language, full-length, peer-reviewed journal articles published from 2019 January 1 through 2026 September 8. The start date defined a focused contemporary review of the period in which matched public multi-omics cohorts and deep, graph-based and attention-based integration became prominent; this review therefore does not claim to cover the historical inception of breast cancer subtyping or multi-omics integration. A study had to include human breast cancer or a multicancer benchmark with a separately reported breast cancer result, use at least 2 molecular omics as actual inputs to the same integrated model, apply machine learning or AI, and predict a prespecified subtype label or explicitly discover or recover patient subtypes. Molecular omics included genomic, transcriptomic, epigenomic, proteomic, metabolomic, or closely related molecular layers. Clinical variables and imaging did not count as a second molecular omic.

Reviews, editorials, abstracts, conference-only papers, preprints, book chapters, non-English reports, and single-omic studies were excluded. Imaging combined with only one molecular omic was ineligible. Studies were also excluded when their only endpoint was diagnosis, tumor stage, prognosis, survival, recurrence, metastasis, drug response, or treatment response. A report with one of those outcomes remained eligible only if it contained a distinct, extractable subtype-prediction or discovery analysis.

### Information sources and search strategy

PubMed/MEDLINE, Europe PMC, and OpenAlex were searched on 2026 September 8. PubMed and Europe PMC were retained as separate, reproducibly exportable biomedical interfaces because their indexing and full-text links are not identical; OpenAlex broadened the coverage of computing and cross-disciplinary journals. Complete queries, fields, limits, and exports are supplied in Tables S1 and S2. PubMed yielded 191 records, Europe PMC 150, and 4 OpenAlex title-and-abstract searches 364 before cross-source deduplication. Backward and forward citation searching of included reports and the 4 closest reviews identified 11 additional reports. Google Scholar was used for targeted searches by title, author, and method name, and IEEE Xplore was consulted for Institute of Electrical and Electronics Engineers (IEEE)-hosted records and full texts. These supplementary routes were reconciled with the saved records and citations rather than treated as independent bulk exports, so they do not add a separate record count to the 705 exported records. Embase, Scopus, and Web of Science were not searched as separate databases; this limitation is considered below.

For every report not obtained during the initial retrieval step, we checked the digital object identifier (DOI) and publisher page, available institutional-access routes, Google Scholar, PubMed, Europe PMC, and OpenAlex; IEEE Xplore was also checked for relevant IEEE-hosted reports. Exact-title and author searches were used to identify legitimate repository copies or author manuscripts. These attempts resolved 21 of the 26 reports that had initially remained outstanding. Five reports could not be obtained in a form suitable for full-report eligibility assessment. Their complete citations, source routes, retrieval attempts, and title/abstract eligibility signals are reported in Table S4; they were not treated as excluded studies. The study-selection process is summarized in Fig. 1.

**Fig. 1.**
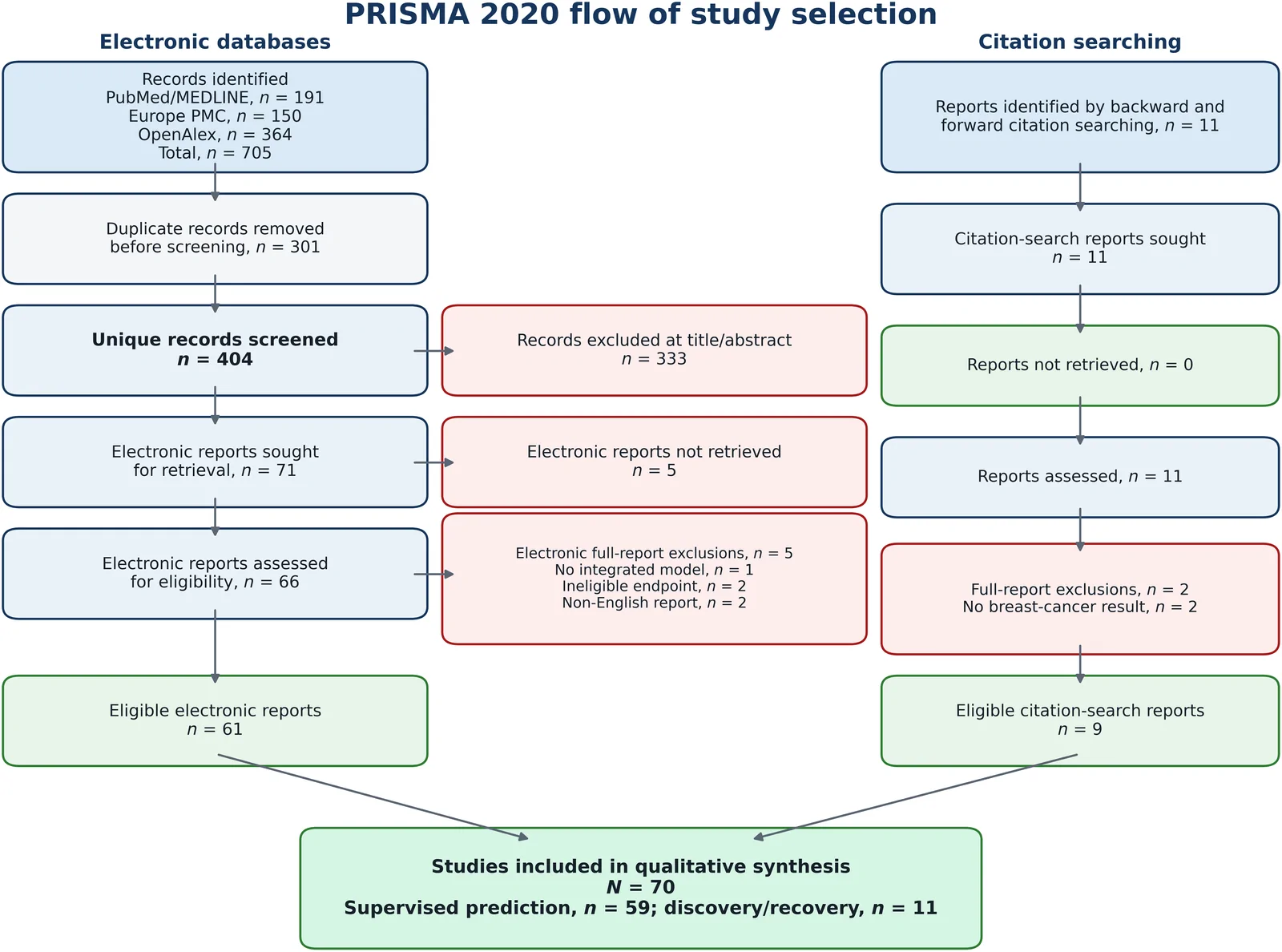
Preferred Reporting Items for Systematic reviews and Meta-Analyses (PRISMA) 2020 flow diagram. Counts are based on saved exports and record-level deduplication. The database and citation-search routes are shown separately until inclusion.

### Record management and study selection

Exports were merged in UTF-8 CSV format. Records were deduplicated first by normalized DOI and then by normalized title; title plus author–year was used when the DOI was absent. One author made the initial title/abstract and full-report eligibility decisions. A second author then checked every completed decision against the eligibility criteria while able to see the first author’s decision and supporting record or report. This was a complete nonblinded verification of the first author’s decisions, not a second blinded *de novo* screening exercise. Disagreements were resolved through discussion and consensus, with a third author available for adjudication if required. Interauthor agreement was not quantified because the verification design did not generate 2 independent blinded rating sets.

### Data extraction

The standardized form captured study identity, cohort, sample size, label source, class structure, omics, preprocessing, missing-data handling, feature selection, fusion stage, representation, interaction mechanism, predictor, attention, validation design, performance metrics, calibration, uncertainty, class imbalance, code availability, explainable AI (XAI), biological plausibility, and external biological replication. One author completed the structured extraction and appraisal. A second author checked every field and judgment against the complete report while able to see the first author’s entries; discrepancies were resolved through discussion and consensus. This was verification of a completed extraction rather than 2 blinded parallel extractions. Publications that reused The Cancer Genome Atlas Breast Invasive Carcinoma (TCGA-BRCA), the Molecular Taxonomy of Breast Cancer International Consortium (METABRIC), or another named resource were counted as separate modeling studies, not as independent patient populations. Development, internal-validation, and external-replication cohorts were therefore mapped at the underlying patient-population level in addition to publication-level counting.

### Orthogonal taxonomy

Five axes were coded. Fusion stage identified where modalities were combined: early, intermediate, late, or hybrid. Representation identified the computational object: tabular/kernel features, learned latent factors, sample or gene graphs, hypergraphs, tensors, topics, or image-transformed omics. Interaction described how information moved between modalities, including concatenation, kernel combination or correlation, joint factorization, message passing, self-attention, cross-attention, and decision aggregation. Predictor identified the final model family. Interpretation distinguished intrinsically interpretable structures, explicit *post hoc* XAI, enrichment or plausibility checks, and external biological replication.

Figure 2 presents the conceptual integration workflows while keeping the axes explicit. Graph, hypergraph, and attention pipelines are not mutually exclusive fusion stages. For example, a method can use intermediate fusion, a patient-graph representation, message passing and attention, a graph neural network (GNN) predictor, and *post hoc* feature attribution simultaneously. The orthogonal taxonomy used for study-level coding is summarized in Fig. 3.

**Fig. 2.**
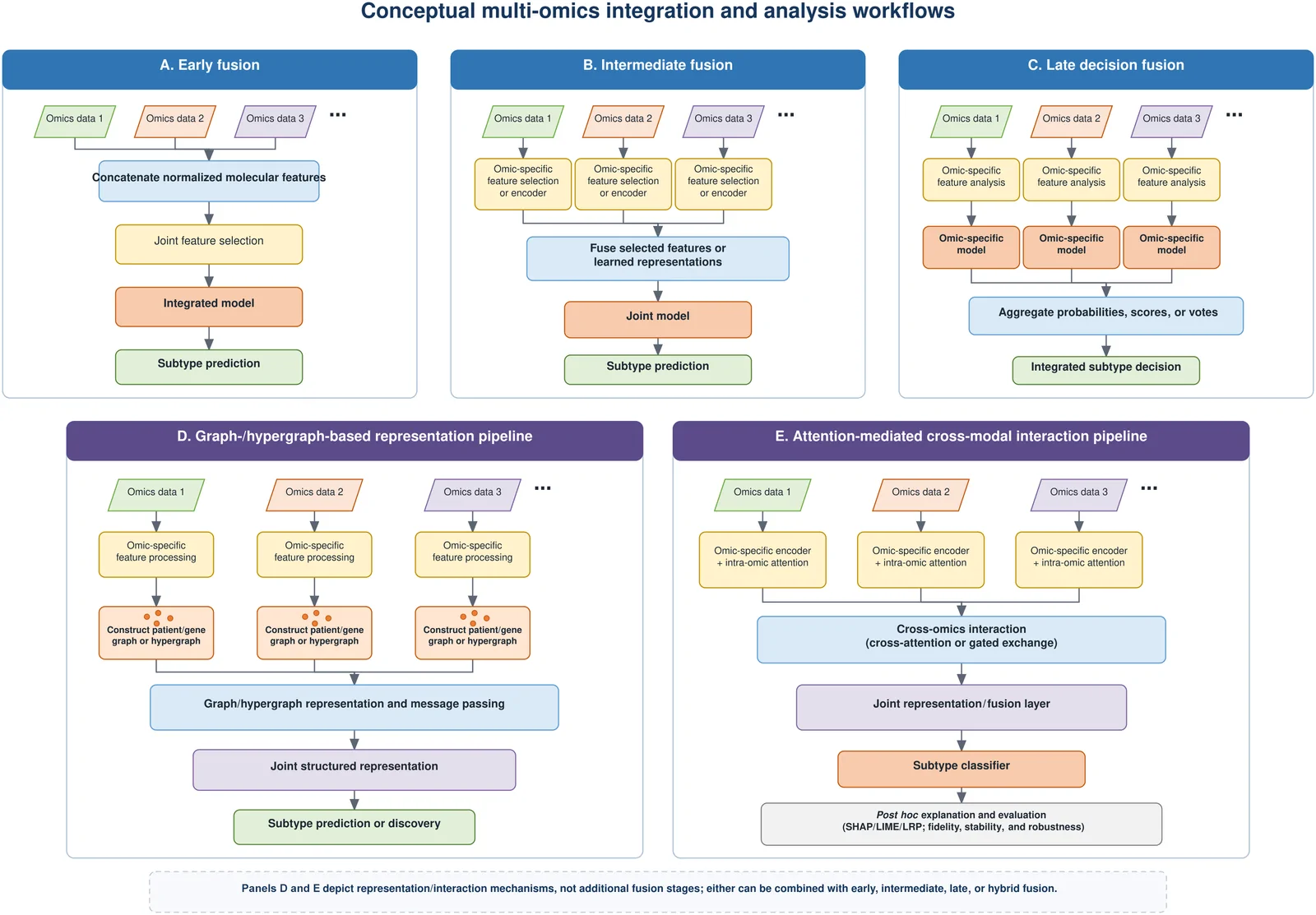
Conceptual workflows for multi-omics integration and analysis. Panels (A) to (C) illustrate early, intermediate, and late decision fusion. Panel (D) illustrates graph- or hypergraph-based structured representation, whereas panel (E) illustrates attention-mediated cross-modal interaction. Panels (D) and (E) are not additional fusion stages and may be implemented within early, intermediate, late, or hybrid fusion architectures.

**Fig. 3.**
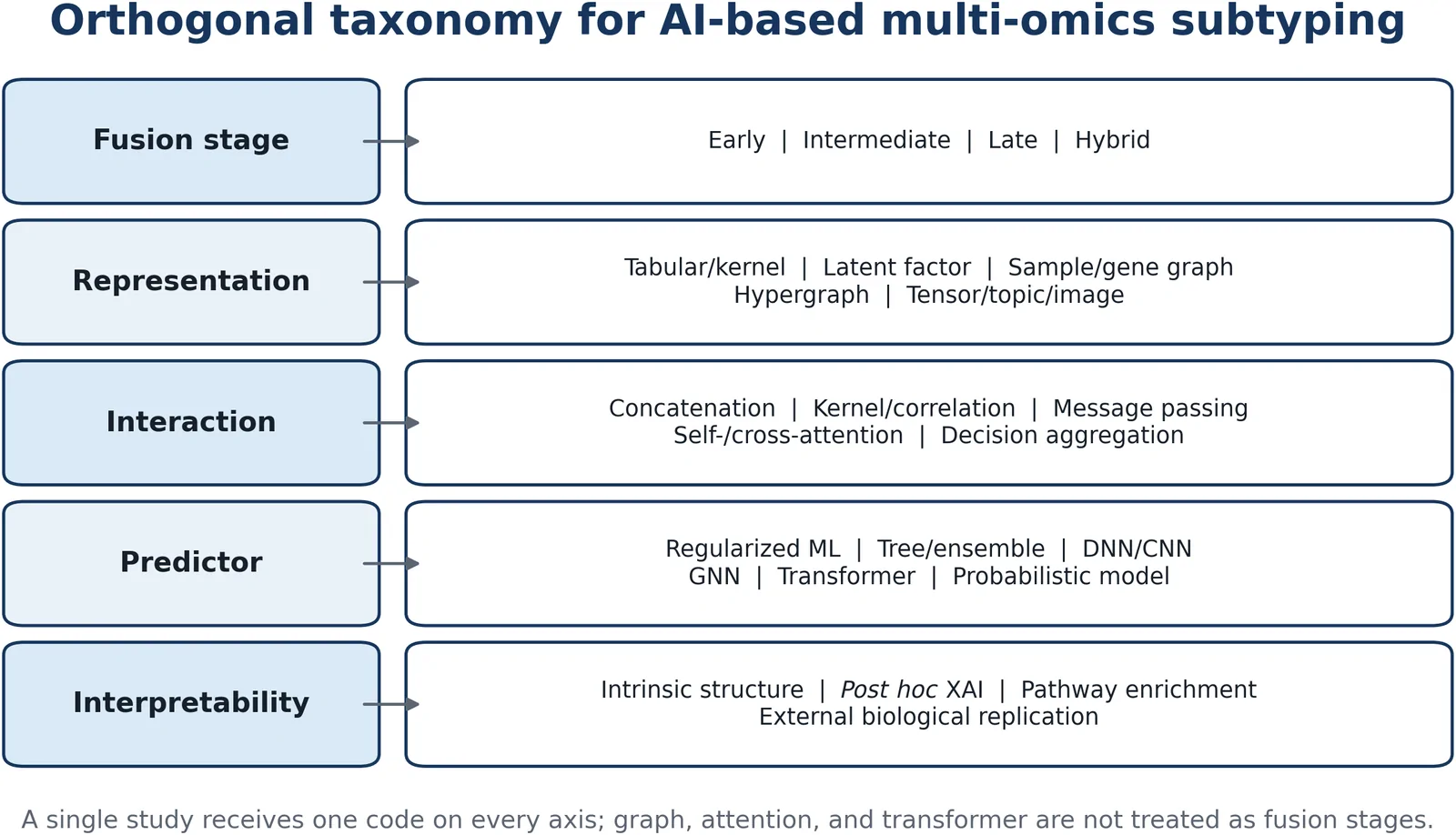
Orthogonal taxonomy used to code the 70 studies. One model can receive one or more codes on each axis; consequently, graph, attention, and fusion-stage counts answer different questions and should not be combined into a single ranking.

### Risk of bias and complementary evidence characteristics

The appraisal kept conceptually different questions separate. For supervised prediction studies, risk of bias was adapted from PROBAST+AI across 4 domains: participants and data, predictors and preprocessing, outcome or reference standard, and analysis [11]. Overall risk of bias was derived only from these domains. Reporting and reproducibility were recorded separately using study identifiers, preprocessing and validation detail, availability of code or models, confidence intervals (CIs), and reproducible data provenance, informed by TRIPOD+AI [12]. Validation and clinical-readiness characteristics were also tabulated separately, including complete external-cohort evaluation, prospective evaluation, nested validation, calibration, uncertainty, class-sensitive metrics, and missing-modality assessment. Interpretability was characterized by explicit XAI, explanation stability/fidelity/robustness, biological plausibility, and independent biological replication.

Data-driven discovery studies were not forced into a supervised prediction risk-of-bias framework. They were assessed using discovery-relevant characteristics: patient and assay selection, preprocessing, cluster or representation stability, sensitivity to analytical choices, biological or clinical association, independent-cohort recovery, and code availability. “Yes” required explicit reporting; absent detail was coded “no/unclear”. Use of transcriptomic predictors for an expression-derived PAM50 endpoint was recorded as reference-standard dependence and limited applicability. It was not treated as automatic information leakage unless the report showed that target information or test-patient information crossed an analytical boundary.

### PAM50 target-dependence and patient-population audits

For studies predicting intrinsic or PAM50-type labels, we additionally recorded whether gene expression was an input, how the target labels were obtained or derived, whether the PAM50 classifier genes and close expression-derived proxies were excluded before model development, whether a transcriptome-only baseline was evaluated on the same patients and data partitions, whether adding nonexpression omics improved a prespecified metric in that matched comparison, and whether the complete multi-omics model was tested in an independent cohort. “Matched incremental value” was assigned only when the report allowed the transcriptome-only and multi-omics results to be compared under the same patient set and evaluation scheme.

To assess population independence, named resources were treated as underlying patient populations rather than as new cohorts each time that a publication created a different subset. TCGA-BRCA-derived benchmark files were mapped to TCGA-BRCA, and METABRIC, GSE22220, AURORA-US Metastasis Project (AURORA, Aiming to Understand the Molecular Aberrations in Metastatic Breast Cancer), GSE84207, GSE202203, GSE96058, and the Netherlands Cancer Institute series were mapped separately. A pooled, resampled, or reprocessed version of a previously used source was not counted as an independent population.

### Synthesis

Studies were grouped first by endpoint and then described chronologically and across the 5 taxonomy axes. Denominators were chosen according to applicability: prediction performance, calibration, imbalance handling, fold containment, nested validation, and external model validation were summarized only for supervised prediction studies; cluster stability and independent recovery were summarized only for discovery studies; and characteristics such as code availability were reported within each endpoint group and, where useful, across the full corpus. “Not applicable” was retained rather than recoded as “no”. No meta-analysis was conducted because label definitions, class counts, cohorts, preprocessing, feature selection, partitioning, tuning, metrics, and averaging conventions differed substantially. Accuracy, F1, area under the receiver operating characteristic curve (AUC), Matthews correlation coefficient (MCC), and clustering measures were retained as author-reported information in the extraction table but were neither pooled nor used to infer architecture-level superiority. Directional statements about multi-omics benefit were limited to matched comparisons within the same study.

## Results

### Study selection

The electronic searches yielded 705 exported records. Deduplication removed 301, leaving 404 unique records. Title/abstract screening excluded 333 records, and 71 electronic reports were sought. Five could not be retrieved after the documented attempts, 66 were assessed, and 5 were excluded after full-report assessment. Citation searching yielded 11 reports; all were retrieved, 11 were assessed, and 2 were excluded. Seventy studies entered the qualitative synthesis: 61 through the database route and 9 through citation searching (Fig. 1). Full-report exclusions and unresolved reports are listed in Tables S3 and S4.

### Evidence landscape: Endpoints, cohorts, and molecular layers

Fifty-seven studies (81.4%) addressed intrinsic or PAM50-type prediction, 11 (15.7%), explicit data-driven discovery or recovery; 1 (1.4%), receptor-status classification; and 1 (1.4%), TNBC versus non-TNBC classification (Table 2). One intrinsic-subtype study also reported ER-status prediction. Keeping these endpoints separate is essential: a binary receptor task can achieve a high AUC under class imbalance, while a 5-class task may fail chiefly in normal-like or HER2-enriched tumors. Discovery studies have no fixed class label at training time and therefore require cluster stability, biological association, and replication rather than only classification accuracy.

**Table 2.** Endpoint strata in the included evidence

Endpoint	Studies, *n*	Percentage, %	Interpretation
Intrinsic/Prediction Analysis of Microarray 50 (PAM50)	57	81.4	Four- or 5-class molecular subtype prediction, often using transcriptomics that overlaps the label-generating assay
Discovery/recovery	11	15.7	Unsupervised or weakly supervised integration intended to identify or recover patient groups
Receptor status	1	1.4	Binary hormone-receptor classification
TNBC status	1	1.4	Binary TNBC versus non-TNBC classification

TNBC, triple-negative breast cancer

Cohort concentration was marked. Sixty-two studies (88.6%) used TCGA-BRCA, and 9 (12.9%) used METABRIC; the categories overlap because some studies used both. Across development, validation, and replication roles, the corpus contained at least 8 named underlying patient populations: TCGA-BRCA, METABRIC, GSE22220, AURORA-US, GSE84207, GSE202203, GSE96058, and the Netherlands Cancer Institute series. Seventy publications therefore do not represent 70 independent cohorts. Repeated cross-validation on a different subset or preprocessing of TCGA-BRCA measures algorithmic variation within the same source population, not population replication. The population-level map is reported in Table S8.

Expression was the near-universal anchor modality. Methylation, miRNA, and copy-number data were commonly paired with it; RPPA and somatic mutation appeared less often. Using more modalities usually reduced the complete-case sample, so apparent biological breadth could be purchased at the cost of statistical power and representativeness. Baena-Miret *et al.* [13] addressed this tension directly with block-wise missing-data modeling; most other studies filtered, imputed, or restricted to overlapping patients.

### Foundations: Kernels, correlations, ensembles, and factor models

The earliest included papers focused on controlling high dimensionality before classification. Tao *et al.* [14] combined mRNA, methylation, and copy number with self-adaptive multiobjective multiple-kernel learning, allowing modality-specific similarity functions to contribute to a joint decision. Qi *et al.* [15] used sparse canonical correlation to capture shared structure across 4 omics and evaluated whether the resulting subtype relationships could be recovered in TCGA-BRCA and GSE22220. ELMO instead trained regularized logistic evidence models for mRNA, miRNA, and methylation and aggregated their evidence, an early example of separating within-modality estimation from cross-modality combination [16].

Tree and forest methods offered nonlinear prediction without end-to-end neural training. Xu *et al.*’s HI-DFNForest [17] first used stacked autoencoders per omic, fused the learned codes with a joint autoencoder, and fed them to a deep flexible neural forest in 104 complete cases. El-Nabawy *et al.* [18] applied a cascade deep forest to expression, miRNA, and copy-number data from METABRIC. These designs illustrate that “deep” integration did not always mean a neural classifier: representation learning could be followed by an ensemble designed for smaller samples.

Other work concentrated on the mathematical object being integrated. MONTI used supervised nonnegative tensor factorization to represent expression, miRNA, and RPPA while incorporating network information [19]. Valle *et al.* [20] developed a multipartite topic model in which cross-omic patterns became latent topics linked to tumor groups. Gygi *et al.* [21] later used supervised Bayesian factors to connect shared mRNA–miRNA variation directly to subtype labels. These approaches yielded lower-dimensional variables that were more structured than raw concatenation, although internal validation still left transportability uncertain.

Chierici *et al.* [22] provided an informative within-study comparison of feature juxtaposition and similarity-network fusion across mRNA, miRNA, copy number, and RPPA, evaluating both PAM50 and ER endpoints and reporting MCC alongside accuracy. aWCluster similarly represented each omic as a patient-distance network but used adaptive network combination for subtype discovery rather than supervised prediction [23]. Together, these papers established 2 recurring ideas: modalities can be integrated through shared patient similarity rather than shared feature space, and the same integration machinery can support either prediction or discovery depending on the endpoint.

The retrieved reports also show that conventional ensembles have remained active alongside deep architectures. TreeEM combined minimum-redundancy maximum-relevance selection with an undersampling random forest and extremely randomized trees for pairwise discrimination among 4 TCGA-BRCA subtypes [24]. This design addressed imbalance directly, but the number of retained variables was selected during the reported cross-validation rather than inside a clearly nested outer evaluation. Its separate mRNA analysis used a different endpoint, so it did not provide a matched transcriptome-only PAM50 baseline.

### Autoencoders and learned latent representations

Autoencoder-based models became a principal intermediate-fusion strategy. Lin *et al.* [25] learned encodings from expression, methylation, and copy number before neural subtype classification; Mohaiminul Islam *et al.* [26] used 4 molecular layers in 1,885 METABRIC tumors; and Khan and Shedole [27] compressed 6 TCGA omics before deep classification. These studies sought a shared signal that was smaller and less redundant than concatenated features. Their main limitation was not representational capacity but evaluation: modality overlap reduced the effective sample size, and internal cross-validation did not show whether the latent space survived cohort or platform change.

CustOmics made the architecture explicitly 2-stage. Source-specific variational autoencoders first captured within-omic structure, and then a central variational model integrated the representations; PAM50 prediction was accompanied by Shapley additive explanations (SHAP) and pathway interpretation [28]. MOCAT used an auxiliary classifier to shape an autoencoder representation from mRNA, miRNA, and RPPA [29]. CAEM-GBDT separated tasks differently: a convolutional autoencoder generated representations from 4 omics, and gradient-boosted trees performed the final prediction [30]. Omran *et al.* [31] compared statistical and deep representations for mRNA–miRNA integration, including MOFA+ factors, and applied SHAP and pathway analysis to the selected model. These designs show that the encoder, fusion operator, and predictor should be assessed separately; an apparent gain cannot automatically be attributed to “deep learning” as one indivisible component.

Moanna provided the strongest validation design in the corpus. A semisupervised autoencoder and multitask network learned from expression, copy number, and mutation in METABRIC, while TCGA-BRCA served as an independent test cohort for the complete pipeline [32]. This is more informative than repartitioning one cohort because it exposes assay, prevalence, and cohort shifts. Nevertheless, one example is insufficient to establish general transportability. OmicsFootPrint pursued a different representation by arranging 5 omics in circular images for deep learning and then mapping SHAP signals back to molecular variables [33]. The approach demonstrated how a structured image can encode multi-omics, but its geometry remains a modeling assumption that requires stability checks.

Recent latent-space models placed more emphasis on evidential decisions and uncertainty. The credal MOD-CINN-CMSC pipeline combined particle filtering, a disentangled graph variational autoencoder, and interval-valued neural decisions for TCGA mRNA and methylation [34]. It reported bootstrap intervals and near-perfect internal results, but the confusion-matrix totals did not reconcile with the stated cohort size, which materially weakens confidence in those estimates. AttentionCCA instead coupled weighted sparse canonical correlation with feature and cross-modal attention [35]. Its TCGA-BRCA analysis included a matched expression-only comparison, whereas the METABRIC experiment transferred only an expression representation and therefore was not counted as external validation of the complete multi-omics model.

### Patient graphs, biological graphs, and hypergraphs

Graph methods expanded rapidly from 2022 onward. MoGCN constructed graph representations from expression, copy number, and RPPA and linked graph-convolutional predictions to enrichment analysis [36]. SUPREME created modality-specific patient-similarity networks, learned graph-convolutional embeddings, and combined embeddings with raw features across TCGA, METABRIC, and a pooled cohort [37]. Multiview graph models used separate omic views before fusion [38], whereas multimodal graph models encoded relationships from expression, miRNA, methylation, and copy number in a shared graph framework [39]. DeepMoIC combined omic-specific autoencoders, similarity-network fusion, and graph convolutional network (GCN) propagation, thereby making representation learning, network integration, and prediction successive steps [40].

AutoGGN was an early gene-graph implementation in the contemporary window. It placed expression and RPPA on a protein-interaction graph, searched GCN/convolutional neural network (CNN) components automatically, and used SHAP to inspect the resulting 4-class model [41]. The attention-based GCN subsequently mapped mRNA, methylation, and copy-number features to a protein-interaction graph; compared row-wise, column-wise, and squeeze-and-excitation attention; and used layer-wise relevance propagation for patient-specific markers [42]. Its reported 5-fold results were strong, but graph construction and all feature processing were not described with enough detail to verify fold containment.

Graph attention added learned neighbor weighting. MOGAT applied attention-weighted message passing to 4 molecular layers and reported macro-F1 for the multiclass BRCA task [43]. MoAGL-SA learned the patient graph rather than fixing it entirely in advance and added self-attention to 3 omics [44]. A semisupervised transformer–GCN design attempted to use structure from unlabeled data as well as feature interactions [45]. In these studies, attention determined how information was combined within a graph; it did not define whether fusion was early or intermediate.

Recent graph systems became more heterogeneous and interpretable. GAIN-BRCA built an omics-associated graph from 5 layers and applied SHAP [46]. AMOGEL connected 4 omics with prior biological knowledge, used integrated gradients, and examined selected biology in an external resource [47]. FastMI-HGNet used 2 streams and heterogeneous relation types with SHAP-based interpretation [48]. Guo *et al.* [49] developed robust multi-omics graph learning and used GNNExplainer, while Siam *et al.* [50] combined modality-specific similarity networks with graph classification and both SHAP and GNNExplainer.

PHyperNET extended pairwise patient graphs to a *p*-Laplacian hypergraph, enabling one hyperedge to represent a higher-order relationship among several samples or features; GNNExplainer was used to inspect the resulting model [51]. The distinction is biologically attractive because molecular programs are rarely pairwise, but it increases design degrees of freedom. Edge construction, similarity thresholds, and graph normalization must all be learned or specified within the training data. If a patient-similarity graph is built once from the entire cohort before cross-validation, test patients can influence the representation available to training patients even without sharing labels.

HyperTMO represented each omic as a patient hypergraph and combined Dirichlet evidence with Dempster–Shafer aggregation, adding predictive uncertainty to a 5-class BRCA analysis [52]. MOFNet used graph pooling and graph convolution within each omic and then fused label-space evidence with a view-correlation network; its report described training-only feature selection, inverse-frequency loss, and an omics ablation [53]. GL-Fusion combined similarity-network graph fusion, structural-entropy optimization, and locus-level graph convolution; RNA was the strongest single layer, while the 3-omic configuration gave the best reported internal result [54]. The initial differential filtering, however, was not clearly confined to training folds.

The 2026 graph studies explored several complementary designs. MuLeCoG used fold-specific analysis of variance filtering, balanced class weights, and hierarchical contrastive learning across 3 patient graphs before support vector machine classification [55]. MCgnn constructed density-aware Mahalanobis graphs, exchanged information through interview attention and fused decisions with view correlation discovery network (VCDN) [56]. MOGANet used one GCN per omic and hierarchical attention to combine the representations, with preprocessing described inside the training data and ablations against single-omic inputs [57]. A PIK3CA-centered analysis used omic-specific GCNs with VCDN or mean fusion and found only a very small macro-F1 difference between its best multi-omics configuration and the matched expression-only baseline [58].

MoACNN-XGNet converted copy number, expression, and methylation into uniform manifold approximation and projection (UMAP) images and classified them with an attention CNN [59]. The reported ablation favored the complete model, and guided gradient-weighted class activation mapping (Grad-CAM) identified prognostic candidates that were examined in independent data. UMAP was nevertheless fitted before cross-validation, allowing held-out patients to influence the representation. The METABRIC analysis was not reported as a locked transfer of the TCGA-trained complete pipeline and was therefore not counted as complete external model validation.

### Attention and transformer-based cross-omics interaction

Attention first appeared as an explicit modality-weighting mechanism in moBRCA-net. Omic-specific branches encoded mRNA, miRNA, methylation, and copy number, after which attention adjusted their contributions before prediction [60]. DMOIT used denoising encoders and multihead self-attention over 4 modalities [61]. These methods can model conditional importance—for example, methylation may matter differently when a transcriptomic program is active—but attention weights alone do not prove faithful explanation.

The 2026 models made cross-modal exchange more explicit. COAT used separate encoders and cross-omics attention among mRNA, miRNA, and methylation, followed by SHAP analysis [62]. GCOA-Net added graph regularization to cross-omics attention, evaluated repeated stratified splits with area under the precision–recall curve (AUPRC) and MCC among its metrics, and used integrated gradients [63]. MOTCS learned a transformer representation from 4 omics across cancer datasets and reported BRCA results with SHAP and biological analysis [64]. A cross-modal self-attention model used 5 METABRIC omics and allowed modality representations to inform one another before classification [65]. These papers reflect a move from one shared bottleneck to explicit pairwise or contextual exchange between modality embeddings.

Two cost-aware models treated uncertainty as part of the fusion decision. Mu *et al.* [66] trained evidential single-view networks and used dynamic Dempster–Shafer fusion to decide whether another modality should be acquired; UMCA-Net combined multistage cross-attention with evidential uncertainty and progressive modality acquisition [67]. Both evaluated missing or deferred modalities, but neither improved BRCA accuracy over its matched mRNA-only configuration. These results are useful because they show that additional assays can improve uncertainty or difficult-case handling without necessarily increasing overall accuracy.

### Subtype discovery, missingness, and special clinical endpoints

The 11 discovery or recovery studies asked whether joint molecular structure could define patient groups rather than only reproduce labels. Sparse correlation [15], adaptive patient networks [23], multipartite topics [20], projection pursuit [68], consensus networks [69], hypergraphs [51], and heterogeneous clustering [70] represented distinct mathematical routes to that goal. The wMKL report was excluded from this group because the full report described applications to prostate and lung adenocarcinoma but no human breast cancer model or separately reported breast cancer result.

Subtype-GAN used a multi-input/multi-output adversarial autoencoder and consensus Gaussian-mixture clustering to identify 5 TCGA-BRCA groups, with subsampling, survival, and clinical associations used to examine their stability and meaning [71]. Viaud *et al.* [72] compared principal component analysis, multiple factor analysis, and several autoencoder designs before *k*-means clustering and showed that the learned representation and source-balancing choices changed the retained cluster structure. Subtype-HM modeled higher-order within- and between-omic relations with multi-level hypergraphs, retained omic-specific information through discriminator-guided attention, and tested stability through repeated 80% subsampling [73].

Wang *et al.* [74] applied a 10-algorithm MOVICS consensus to 6 TCGA-BRCA omics and selected 5 groups with distinct survival, immune, and molecular characteristics. Leave-one-omic-out analyses retained more than 85% concordance, and expression-derived subtype signatures were examined in GSE202203, GSE96058, METABRIC, and the Netherlands Cancer Institute series. Together with transfer to GSE22220 [15] and biological examination in AURORA-US [68], this was 1 of only 3 discovery programs attempting recovery in an independent patient population. In discovery work, agreement with PAM50 is informative but not sufficient: a credible novel subtype should also be stable under resampling, associated with coherent biology or outcome, and recoverable in independent samples.

Missing modalities were usually treated as a preprocessing inconvenience. Baena-Miret *et al.* [13] instead designed a 2-step algorithm for block-wise missing expression and RPPA; the dynamic evidential framework and UMCA-Net evaluated progressive acquisition or missing-modality operation [66,67]. These 3 studies addressed the realistic situation in which an entire assay is unavailable or unnecessary for some patients. Models requiring all input layers at deployment can be clinically unusable even if complete-case accuracy is high. Future evaluations should distinguish random element-wise missingness, block-wise assay absence, and systematic missingness related to site or patient characteristics.

Only 2 studies were centered on special clinical labels. Yang *et al.* [75] used 5 molecular omics in a deep model for TNBC versus non-TNBC classification. Unlu Yazici *et al.* [76] applied multi-omics feature grouping and regularized learning to hormone-receptor-positive versus hormone-receptor-negative tumors. Their binary endpoints are clinically recognizable, but they do not measure the same problem as 4- or 5-class intrinsic prediction and were not compared numerically with PAM50 studies.

### Contribution of included studies

Table 3 provides a chronological account of all 70 studies. It is intentionally more descriptive than a conventional characteristics table: the final column identifies the main integration step or methodological contribution. Detailed preprocessing, validation, reported outcomes, and the separated appraisal domains are provided in Tables S5 to S7.

**Table 3.** Study-by-study account of the included literature

Study	Cohort and endpoint	Molecular inputs	What the study did
Tao *et al.* (2019)	TCGA-BRCA; intrinsic/Prediction Analysis of Microarray 50 (PAM50)	mRNA; DNA methylation; copy-number alteration	Combined gene expression, DNA methylation, and copy-number data through a self-adaptive multiobjective multiple-kernel learning strategy. The study used pathway-informed feature filtering and evaluated receptor-defined and intrinsic-subtype classification in TCGA-BRCA.
Xu *et al.* (2019)	TCGA-BRCA; intrinsic/PAM50	mRNA; miRNA; DNA methylation	Introduced HI-DFNForest: separate stacked autoencoders compressed mRNA, miRNA, and methylation; a joint autoencoder fused the modality representations; and a deep flexible neural forest performed classification in 104 complete TCGA-BRCA cases.
Chierici *et al.* (2020)	TCGA-BRCA; intrinsic/PAM50	mRNA; miRNA; copy-number alteration; protein/RPPA	Compared feature juxtaposition with similarity-network fusion for mRNA, miRNA, copy-number, and RPPA. Models were assessed for PAM50 and ER status using 5-fold cross-validation, with MCC and class-specific measures supplementing accuracy.
Lin *et al.* (2020)	TCGA-BRCA; intrinsic/PAM50	mRNA; DNA methylation; copy-number alteration	Learned modality-specific encodings from expression, methylation, and copy-number profiles; joined the latent information and used a deep neural network to predict intrinsic subtypes in TCGA-BRCA.
Mohaiminul Islam *et al.* (2020)	METABRIC; intrinsic/PAM50	mRNA; DNA methylation; copy-number alteration; protein/RPPA	Developed an integrative deep-learning pipeline on 1,885 METABRIC tumors, combining expression, methylation, copy-number, and protein information after feature filtering to classify breast cancer subtypes.
Zhang *et al.* (2020)	TCGA-BRCA; intrinsic/PAM50	mRNA; miRNA; DNA methylation	Proposed ELMO, a regularized logistic evidence framework that produced evidence from mRNA, miRNA, and methylation models and aggregated it for TCGA-BRCA subtype prediction.
El-Nabawy *et al.* (2021)	METABRIC; intrinsic/PAM50	mRNA; miRNA; copy-number alteration	Applied a cascade deep-forest ensemble to METABRIC multi-omics. Expression, miRNA, and copy-number inputs were filtered and integrated in a tree-based model intended to remain effective with limited sample size.
Jung *et al.* (2021)	TCGA-BRCA; intrinsic/PAM50	mRNA; miRNA; protein/RPPA	Introduced MONTI, a supervised nonnegative tensor-factorization model that represented gene expression, miRNA, and RPPA jointly; incorporated network information; and used learned factors for subtype classification.
Qi *et al.* (2021)	TCGA-BRCA; GSE22220; data-driven discovery/recovery	mRNA; miRNA; DNA methylation; copy-number alteration	Used sparse CCA to link mRNA, miRNA, methylation, and copy-number measurements; recover multi-omics subtype structure; and examine transfer between TCGA-BRCA and GSE22220.
Zhang *et al.* (2021)	TCGA-BRCA; intrinsic/PAM50	mRNA; protein/RPPA	Combined a protein-interaction gene graph with expression and RPPA through an automatically configured GCN/CNN pipeline. The model used repeated cross-validation plus a hold-out test and applied SHAP to influential molecular features.
Yang *et al.* (2021)	TCGA-BRCA; data-driven discovery/recovery	mRNA; miRNA; DNA methylation; copy-number alteration	Learned a shared adversarial latent space from 4 omics and used consensus Gaussian-mixture clustering to identify 5 subtypes. Subsampling, survival, and clinical associations were used to examine the clusters.
Khan and Shedole (2022)	TCGA-BRCA; intrinsic/PAM50	mRNA; miRNA; DNA methylation; copy-number alteration; somatic mutation; protein/RPPA	Compressed 6 TCGA molecular layers with autoencoder-based representation learning and classified PAM50 groups with a deep neural network, illustrating both the potential and complete-case cost of high-modality integration.
Li *et al.* (2022)	TCGA-BRCA; intrinsic/PAM50	mRNA; copy-number alteration; protein/RPPA	Proposed MoGCN, constructing omics-informed graph representations and applying graph convolution to mRNA, copy number, and RPPA. The study linked predictive evaluation with enrichment and biologically relevant feature interpretation.
Meshoul *et al.* (2022)	TCGA-BRCA; intrinsic/PAM50	mRNA; miRNA; DNA methylation; copy-number alteration; protein/RPPA	Built an explainable multiclass framework from 5 TCGA omics using statistical filtering and regularized classifiers and then used SHAP to attribute subtype predictions to molecular variables.
Pouryahya *et al.* (2022)	TCGA-BRCA; data-driven discovery/recovery	mRNA; DNA methylation; copy-number alteration	Developed aWCluster for subtype discovery by turning expression, methylation, and copy-number measurements into patient-distance networks and adaptively combining them before cluster assessment.
Valle *et al.* (2022)	TCGA-BRCA; data-driven discovery/recovery	mRNA; miRNA; copy-number alteration	Represented expression, miRNA, and copy-number data as a multipartite topic model. The inferred topics provided a joint low-dimensional description used to recover tumor groups and molecular associations.
Viaud *et al.* (2022)	TCGA-BRCA; data-driven discovery/recovery	DNA methylation; miRNA; mRNA; protein/RPPA	Compared PCA, multiple factor analysis, and stacked or disjoint autoencoders before *k*-means clustering. A weighted internal index assessed whether the representation retained structure from each omic, and sensitivity analyses varied cluster number and architecture.
Benkirane *et al.* (2023)	TCGA-BRCA; intrinsic/PAM50	mRNA; miRNA; DNA methylation; copy-number alteration; protein/RPPA	Presented CustOmics, a 2-phase architecture with source-specific variational autoencoders followed by a central variational model. Five omics were integrated for PAM50 prediction, and SHAP and pathway analyses were used to inspect the learned signal.
Choi and Chae (2023)	TCGA-BRCA; intrinsic/PAM50	mRNA; miRNA; DNA methylation; copy-number alteration	Developed moBRCA-net with omic-specific branches and an attention module that weighted information across mRNA, miRNA, methylation, and copy number before multiclass prediction.
Kesimoglu and Bozdag (2023)	TCGA-BRCA; METABRIC; combined TCGA plus METABRIC; intrinsic/PAM50	mRNA; miRNA; DNA methylation; copy-number alteration	Introduced SUPREME, creating a patient-similarity network for each modality, learning graph-convolutional embeddings and combining those embeddings with raw features. It evaluated TCGA-BRCA, METABRIC, and a combined cohort.
Lupat *et al.* (2023)	METABRIC development; TCGA-BRCA external test; intrinsic/PAM50	mRNA; copy-number alteration; somatic mutation	Proposed Moanna, a semisupervised autoencoder and multitask network using expression, copy number, and mutation. The complete model was developed in METABRIC and tested in TCGA-BRCA, making it the sole cross-cohort full-model validation in this review.
Unlu Yazici *et al.* (2023)	TCGA-BRCA; intrinsic/PAM50	mRNA; miRNA; DNA methylation; copy-number alteration; protein/RPPA	Introduced 3Mint, which grouped and scored features across 5 omics before regularized modeling. The analysis focused on separating luminal and ER-negative disease and connected selected variables with biological interpretation.
Guo *et al.* (2023)	TCGA-BRCA; intrinsic/PAM50	mRNA; DNA methylation; copy-number alteration	Mapped 3 gene-level omics to a protein-interaction graph, compared several attention mechanisms, and used layer-wise relevance propagation to identify patient-specific markers. Five-fold cross-validation reported accuracy, AUC, and MCC.
Cheng *et al.* (2024)	TCGA-BRCA; intrinsic/PAM50	mRNA; miRNA; DNA methylation	Proposed MoAGL-SA, coupling adaptive patient-graph learning with self-attention across expression, miRNA, and methylation to classify molecular subtypes and identify biologically associated signals.
Gygi *et al.* (2024)	TCGA-BRCA; intrinsic/PAM50	mRNA; miRNA	Used a supervised Bayesian latent-factor model to integrate mRNA and miRNA while linking the shared representation directly to subtype labels, providing probabilistic dimension reduction rather than deterministic concatenation.
Huang *et al.* (2024)	TCGA-BRCA; intrinsic/PAM50	mRNA; DNA methylation	Combined sparse canonical-correlation feature extraction from expression and methylation with a downstream deep model, using cross-omic covariance to reduce dimensionality before PAM50 classification.
Li and Nabavi (2024)	TCGA-BRCA; intrinsic/PAM50	mRNA; miRNA; DNA methylation; copy-number alteration	Constructed a multimodal GNN from expression, miRNA, methylation, and copy-number information. Omics-derived relationships were propagated through the graph for subtype classification.
Liu and Park (2024)	TCGA-BRCA; intrinsic/PAM50	mRNA; DNA methylation; copy-number alteration; protein/RPPA	Introduced DMOIT, a denoising multi-omics integration transformer that encoded 4 molecular layers and used multihead self-attention to learn cross-feature dependencies before subtype prediction.
Ren *et al.* (2024)	TCGA-BRCA; intrinsic/PAM50	mRNA; DNA methylation; copy-number alteration; protein/RPPA	Developed a multiview GNN in which 4 omics generated complementary graph views; graph representations were fused and evaluated by 5-fold cross-validation for intrinsic-subtype prediction.
Shen *et al.* (2024)	Public breast cancer cohort (name not clear); intrinsic/PAM50	mRNA; miRNA; DNA methylation; protein/RPPA	Combined convolutional autoencoder representations of expression, miRNA, methylation, and RPPA with gradient-boosted decision trees, separating nonlinear feature construction from the final classifier.
Tang *et al.* (2024)	TCGA-BRCA; intrinsic/PAM50	mRNA; miRNA; copy-number alteration; somatic mutation; protein/RPPA	Presented OmicsFootPrint, mapping 5 omics into structured circular images for deep learning. SHAP and downstream biological analyses were used to connect image regions back to molecular features.
Tanvir *et al.* (2024)	TCGA-BRCA; intrinsic/PAM50	mRNA; miRNA; DNA methylation; copy-number alteration	Proposed MOGAT, a graph-attention model that integrated 4 molecular layers and used attention-weighted message passing for PAM50 classification; the study reported macro-F1 to reflect multiclass performance.
Wang *et al.* (2024)	Public breast cancer cohort (name not clear); intrinsic/PAM50	mRNA; miRNA; DNA methylation; copy-number alteration; protein/RPPA	Combined a semisupervised transformer with graph convolution over 5 omics, aiming to use unlabeled structures while modeling both long-range feature relations and graph neighborhood information.
Wu *et al.* (2024)	TCGA-BRCA; intrinsic/PAM50	mRNA; miRNA; DNA methylation; copy-number alteration; protein/RPPA	Developed DeepMoIC with omic-specific autoencoders, similarity-network fusion, and graph convolution. Five TCGA omics were reduced, integrated as a patient network, and examined through biological enrichment.
Yang *et al.* (2024)	TCGA-BRCA; TCIA imaging subset; TNBC status	mRNA; miRNA; DNA methylation; copy-number alteration; somatic mutation	Integrated 5 molecular omics at the feature level in a deep neural network to distinguish TNBC from non-TNBC, a binary endpoint kept separate from PAM50 studies in this review.
Yao *et al.* (2024)	Public breast cancer cohort (name not clear); intrinsic/PAM50	mRNA; miRNA; protein/RPPA	Proposed MOCAT, using an auxiliary-classifier-enhanced autoencoder to learn a joint representation from expression, miRNA, and RPPA before intrinsic-subtype prediction.
Zeng *et al.* (2024)	TCGA-BRCA; intrinsic/PAM50	mRNA; miRNA; DNA methylation	Developed DiffRS-net, which selected informative variables from expression, miRNA, and methylation and compared machine-learning classifiers for multiclass subtype prediction.
Zhang *et al.* (2024)	TCGA-BRCA; intrinsic/PAM50	mRNA; DNA methylation; protein/RPPA	Presented AET-net, integrating expression, methylation, and RPPA with representation and network-informed feature selection; class balancing was incorporated before subtype classification.
Wang *et al.* (2024)	TCGA-BRCA; intrinsic/PAM50	mRNA; DNA methylation; miRNA	Represented each omic as a patient hypergraph and used hypergraph convolution followed by Dirichlet evidence and Dempster–Shafer fusion. The model reported uncertainty alongside accuracy and macro/weighted F1.
Baena-Miret *et al.* (2025)	TCGA-BRCA; intrinsic/PAM50	mRNA; protein/RPPA	Studied block-wise missing multi-omics using a 2-step strategy for expression and RPPA, allowing subtype prediction when some patients lacked an entire modality rather than restricting analysis to complete cases.
Bong *et al.* (2025)	TCGA-BRCA; AURORA-US (biological replication); data-driven discovery/recovery	mRNA; DNA methylation; protein/RPPA	Applied augmented projection pursuit using kurtosis to find non-Gaussian multi-omics structure in expression, methylation, and RPPA. Clusters learned in TCGA were biologically examined in AURORA transcriptomes.
Hu *et al.* (2025)	TCGA-BRCA; data-driven discovery/recovery	mRNA; miRNA; copy-number alteration; protein/RPPA	Built a consensus network approach that integrated expression, miRNA, copy number, and RPPA to map intertumor heterogeneity, identify subtype structure, and examine biological associations.
Mukhdoomi and Chachoo (2025)	TCGA-BRCA; data-driven discovery/recovery	mRNA; miRNA; DNA methylation; copy-number alteration; protein/RPPA	Introduced PHyperNET, representing 5 omics with a *p*-Laplacian hypergraph so that higher-order patient relations, rather than only pairwise edges, could contribute to subtype discovery; GNNExplainer was used *post hoc*.
Omran *et al.* (2025)	TCGA-BRCA; intrinsic/PAM50	mRNA; miRNA	Compared statistical and deep representation strategies for mRNA–miRNA integration, including MOFA+-derived factors followed by nonlinear classification. SHAP and pathway analysis were used to inspect the selected representation.
Patel *et al.* (2025)	TCGA-BRCA; intrinsic/PAM50	mRNA; miRNA; DNA methylation; copy-number alteration; protein/RPPA	Proposed GAIN-BRCA, constructing an omics-associated graph from 5 molecular layers and applying a graph model for intrinsic-subtype prediction, with SHAP used to attribute predictions.
Pateras *et al.* (2025)	TCGA-BRCA; data-driven discovery/recovery	mRNA; miRNA; DNA methylation; copy-number alteration; protein/RPPA	Used heterogeneous multi-omics clustering across 5 data types to recover breast cancer groups and relate the resulting structure to established molecular subtypes and pathway-level biology.
Tan *et al.* (2025)	TCGA-BRCA; intrinsic/PAM50	mRNA; miRNA; DNA methylation; protein/RPPA	Developed AMOGEL, an associative GNN that combined 4 omics with prior biological knowledge. Integrated gradients identified influential features, and selected biology was examined in an external resource.
Unlu Yazici *et al.* (2025)	TCGA-BRCA; receptor status	mRNA; miRNA; DNA methylation; protein/RPPA	Extended the 3Mint strategy to HR status using 4 omics, scoring feature groups before regularized classification of HR-positive and HR-negative tumors and examining selected biological variables.
Zhao and Li (2025)	TCGA-BRCA; intrinsic/PAM50	Copy-number alteration; mRNA; protein/RPPA	Combined MRMR selection, an undersampling random forest, and extremely randomized trees for pairwise classification among 4 subtypes. The feature dimension was selected by internal cross-validation rather than a reported nested outer loop.
Zhang *et al.* (2025)	TCGA-BRCA; intrinsic/PAM50	mRNA; DNA methylation; miRNA	Learned omic-specific similarity graphs, pooled features, and fused label-space evidence with view correlation discovery network (VCDN). The report described training-only preprocessing, inverse-frequency loss, stratified splits, and omics ablation.
Tan *et al.* (2025)	TCGA-BRCA; intrinsic/PAM50	mRNA; DNA methylation; somatic copy-number variation	Fused gene graphs using similarity-network fusion and structural entropy and then combined locus-aligned features in a GCN. RNA was the strongest single layer, and the 3-omic model was strongest in the reported ablation.
Atfa *et al.* (2026)	TCGA-BRCA; intrinsic/PAM50	mRNA; miRNA; DNA methylation	Introduced COAT, an encoder-based cross-omics attention transformer for mRNA, miRNA, and methylation. It modeled directed interactions between modality representations and used SHAP for feature attribution.
Fu *et al.* (2026)	TCGA-BRCA; intrinsic/PAM50	mRNA; miRNA; DNA methylation; protein/RPPA	Proposed FastMI-HGNet, a 2-stream heterogeneous graph network for expression, miRNA, methylation, and RPPA, with message passing across distinct relation types and SHAP-based interpretation.
Guo *et al.* (2026)	TCGA-BRCA; intrinsic/PAM50	mRNA; miRNA; copy-number alteration; somatic mutation; protein/RPPA	Developed a robust multi-omics graph-learning framework using 5 molecular layers in 450 PAM50-labeled cases. It combined representation learning and message passing and used GNNExplainer to inspect graph predictions.
Li *et al.* (2026)	TCGA-BRCA; intrinsic/PAM50	mRNA; miRNA; DNA methylation	Presented GCOA-Net, combining graph regularization with cross-omics attention for expression, miRNA, and methylation. Repeated stratified validation included macro metrics and AUPRC, and integrated gradients supported attribution.
Meng *et al.* (2026)	TCGA-BRCA; intrinsic/PAM50	mRNA; miRNA; DNA methylation; protein/RPPA	Introduced MOTCS, a transformer-based multi-omics classifier that learned a shared representation from 4 modalities across cancer datasets, reported BRCA results separately, and applied SHAP with biological analysis.
Siam *et al.* (2026)	Public breast cancer cohort (name not clear); intrinsic/PAM50	mRNA; miRNA; DNA methylation; protein/RPPA	Modeled modality-specific patient-similarity networks from 4 omics and used graph classification for breast cancer subtype prediction; SHAP and GNNExplainer were applied to tabular and graph components.
Zhumagozhayev *et al.* (2026)	METABRIC; intrinsic/PAM50	mRNA; miRNA; DNA methylation; copy-number alteration; somatic mutation; protein/RPPA	Evaluated a hybrid machine-learning pipeline on 1,523 METABRIC cases using 6 omics, statistical and embedded feature selection, and layer-wise relevance propagation to identify subtype-associated variables.
Zhumagozhayev *et al.* (2026)	METABRIC; intrinsic/PAM50	mRNA; miRNA; DNA methylation; copy-number alteration; somatic mutation	Developed a cross-modal self-attention model on 1,523 METABRIC tumors, with separate encoders for 5 omics and attention-mediated exchange between modality representations before subtype classification.
Yang *et al.* (2026)	TCGA-BRCA; intrinsic/PAM50	mRNA; DNA methylation; miRNA	Built one patient graph per omic and aligned local and cross-view representations through hierarchical contrastive learning before SVM classification. ANOVA selection and tuning were described within training folds, with balanced class weights.
Mu *et al.* (2026)	TCGA-BRCA; intrinsic/PAM50	mRNA; DNA methylation; miRNA	Trained evidential single-view networks and used dynamic Dempster–Shafer fusion to acquire modalities progressively. It quantified uncertainty, but the full tri-omics BRCA accuracy did not exceed the matched mRNA-only result.
Zhang *et al.* (2026)	TCGA-BRCA; METABRIC expression-only transfer; intrinsic/PAM50	mRNA; DNA methylation; miRNA	Extended sparse CCA with feature and cross-modal attention. The TCGA ablation favored multi-omics, while the METABRIC experiment used only expression and was not a test of the complete multi-omics pipeline.
Subramaniam and Manoharan (2026)	TCGA-BRCA; intrinsic/PAM50	mRNA; DNA methylation	Combined particle filtering, a disentangled graph variational autoencoder, and a credal interval classifier. Bootstrap intervals were reported, but sample totals in the confusion matrices did not reconcile with the stated cohort size.
Wang *et al.* (2026)	TCGA-BRCA; GSE202203; GSE96058; METABRIC; NKI; data-driven discovery/recovery	mRNA; miRNA; lncRNA; DNA methylation; copy-number alteration; somatic mutation	Applied a 10-algorithm MOVICS consensus to identify 5 groups, tested leave-one-omic sensitivity, and evaluated expression-derived subtype recovery in 4 independent cohorts, alongside survival, immune, and drug analyses.
Li *et al.* (2026)	TCGA-BRCA; intrinsic/PAM50	mRNA; DNA methylation; miRNA	Constructed density-aware Mahalanobis patient graphs, used stacked graph convolution and inter-view attention, and fused decisions with VCDN. Masking-based scores supported biomarker and enrichment analyses.
Wu *et al.* (2026)	TCGA-BRCA; intrinsic/PAM50	mRNA; DNA methylation; miRNA	Used one GCN per omic and hierarchical attention to combine within- and between-modality information. The report described training-only preprocessing and compared single-, dual-, and 3-omic configurations.
Li *et al.* (2026)	TCGA-BRCA; METABRIC; GSE84207; intrinsic/PAM50	Copy-number alteration; mRNA; DNA methylation	Converted 3 omics to UMAP images and classified them with an attention CNN. Guided Grad-CAM supported gene analysis, but UMAP before cross-validation introduced leakage concern and locked cross-cohort transfer was unclear.
Wang *et al.* (2026)	TCGA-BRCA; data-driven discovery/recovery	mRNA; miRNA; DNA methylation; copy-number alteration	Modeled higher-order within- and between-omic relations with multi-level hypergraphs, retained modality-specific information through attention, and aligned subtype assignments using contrastive entropy. Stability was tested in repeated subsamples.
Huang *et al.* (2026)	TCGA-BRCA; intrinsic/PAM50	mRNA; DNA methylation; miRNA	Combined multistage cross-attention with evidential uncertainty and cost-aware progressive modality acquisition. The method handled missing modalities, although tri-omics BRCA accuracy did not exceed mRNA alone.
Lan *et al.* (2026)	TCGA-BRCA; intrinsic/PAM50	mRNA; DNA methylation; miRNA	Applied omic-specific GCNs with VCDN and mean fusion in a PIK3CA-centered study. The matched BRCA comparison showed only a very small macro-F1 difference between the best fusion and mRNA alone.

ANOVA, analysis of variance; AUC, area under the receiver operating characteristic curve; AUPRC, area under the precision–recall curve; AURORA, Aiming to Understand the Molecular Aberrations in Metastatic Breast Cancer; CCA, canonical correlation analysis; CNN, convolutional neural network; ER, estrogen receptor; GCN, graph convolutional network; GNN, graph neural network; Grad-CAM, gradient-weighted class activation mapping; HR, hormone receptor; lncRNA, long noncoding RNA; MCC, Matthews correlation coefficient; METABRIC, Molecular Taxonomy of Breast Cancer International Consortium; miRNA, microRNA; MRMR, minimum-redundancy maximum-relevance; mRNA, messenger RNA; PCA, principal component analysis; RPPA, reverse-phase protein array; SHAP, Shapley additive explanations; SVM, support vector machine; TCGA-BRCA, The Cancer Genome Atlas Breast Invasive Carcinoma; TCIA, The Cancer Imaging Archive; TNBC, triple-negative breast cancer; UMAP, uniform manifold approximation and projection

### Fusion, representation, and model-family patterns

Intermediate fusion was coded in 43/70 studies (61.4%); hybrid fusion, in 20/70 (28.6%); early fusion, in 5/70 (7.1%); and late fusion, in 2/70 (2.9%). On the representation axis, 18 studies primarily used tabular or kernel forms, 22 used learned latent representations, 23 used sample or gene graphs, 3 used hypergraphs, 2 used image-transformed representations, 1 used a tensor, and 1 used a topic model. These groupings identify the principal representation in each study and describe frequency, not effectiveness.

Early integration is simple and permits conventional feature selection, as in the TNBC and receptor-status studies [75,76], but a high-dimensional modality can dominate concatenation and block-wise missingness is awkward. Intermediate integration preserves modality-specific processing and was used by autoencoders, factor models, similarity networks, graphs, and transformers. Hybrid designs combined more than one stage, for example, juxtaposing features with network fusion [22] or joining network embeddings and raw features [37]. The corpus contained no clean test of late decision fusion against otherwise identical alternatives, so its apparent rarity should not be mistaken for inferiority.

### Validation, performance reporting, and risk of bias

Only Moanna trained a complete model in one named cohort and evaluated the locked multi-omics pipeline in another [32]. All remaining supervised studies relied on splits or cross-validation within the same source cohort, transferred only part of the pipeline, or did not describe validation clearly enough to establish independence. None of the 59 supervised studies reported prospective evaluation or nested validation of the full feature-selection, fusion, and tuning procedure. Eleven (18.6%) explicitly kept learned preprocessing or feature selection within training folds, 12 (20.3%) addressed class imbalance, 8 (13.6%) reported CIs, 1 (1.7%) reported calibration, 4 (6.8%) quantified predictive uncertainty, 3 (5.1%) evaluated missing or progressively acquired modalities, and 34 (57.6%) made code or software available.

Overall risk of bias was high in 48/59 supervised studies and raised some concerns in 11/59; none met the criteria for low overall risk. In the analysis domain, 43/59 were high-risk, 15/59 raised some concerns, and 1 was low-risk. Predictors and preprocessing were high-risk in 48/59 and raised some concerns in 11/59, whereas participant/data judgments were high-risk in 1 study and raised some concerns in 58. These judgments do not imply that every reported result is incorrect. They identify conditions that could inflate performance or limit applicability, particularly preprocessing outside resampling, extensive model selection without an outer validation loop, small rare-subtype groups, and absence of external evaluation. Reporting, reproducibility, interpretability, and clinical-readiness characteristics were not used to determine the overall risk-of-bias judgment.

The PAM50 audit sharpened a separate applicability concern. All 57 intrinsic/PAM50 studies used gene expression, and 36/57 (63.2%) clearly documented the origin of the target labels. None reported excluding the PAM50 classifier genes or close expression-derived proxies before model development. Thirty-nine (68.4%) evaluated a matched expression-only baseline, and 35/57 (61.4%) reported improvement in at least one prespecified metric after adding nonexpression omics under that study’s matched comparison. These author-reported gains are useful within-study evidence but do not establish statistical significance, clinical benefit, or transportability; only 1 of the 57 tested the complete multi-omics pipeline in an independent cohort.

The 11 discovery studies required different evidence. Four assessed stability under resampling or perturbation, 5 examined sensitivity to analytical choices, 8 related the groups to biological or clinical characteristics, 3 attempted recovery in an independent patient population, and 6 made code available. Thus, biological association was more common than direct evidence that the same grouping can be recovered when the population or assay setting changes. Figure 4 displays the prediction and discovery denominators separately.

**Fig. 4.**
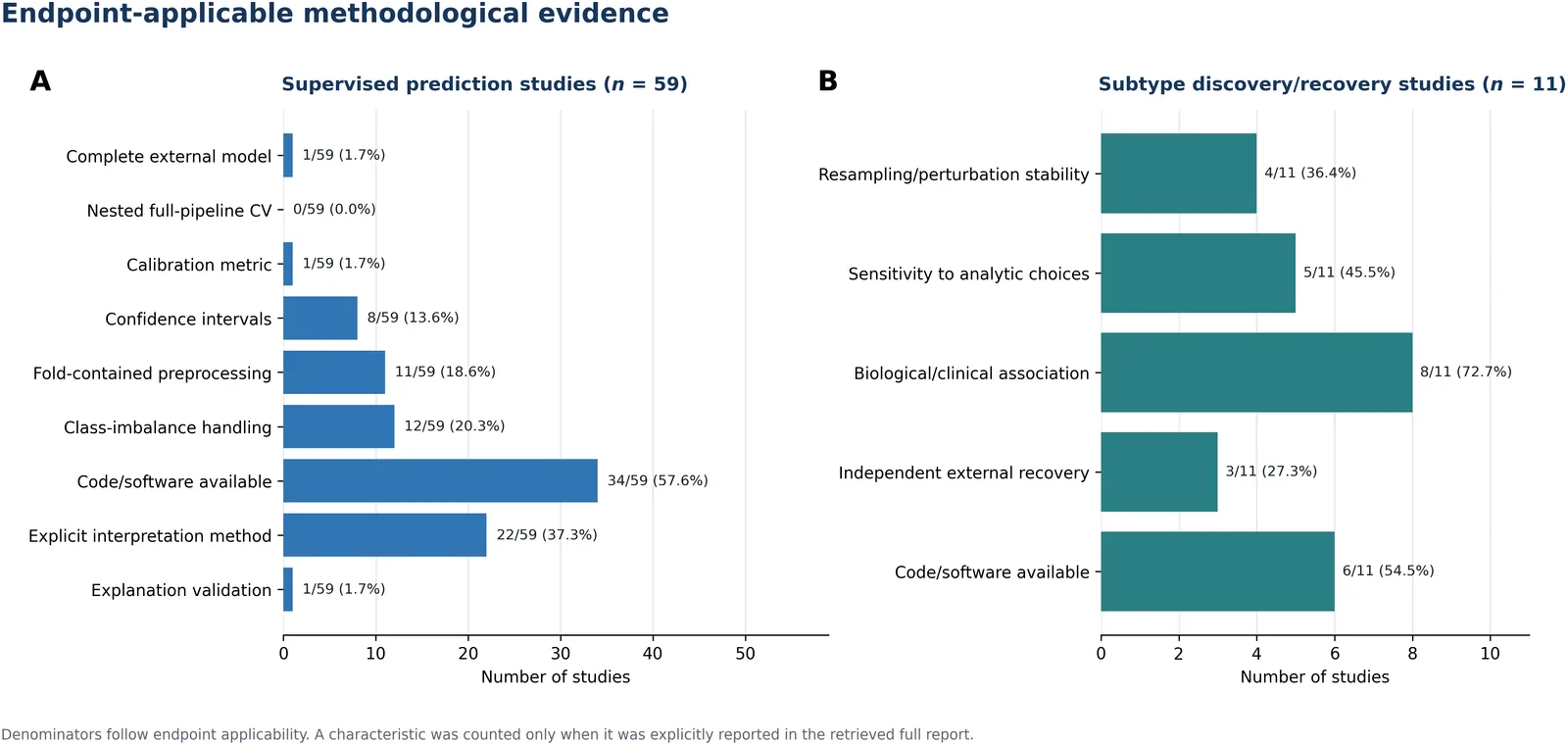
Endpoint-specific evidence profile. Panel (A) summarizes supervised prediction studies (*n* = 59). Panel (B) summarizes subtype discovery or recovery studies (*n* = 11). Counts use conservative explicit-reporting rules. “Not reported” does not prove that a procedure was not performed.

Author-reported accuracy, F1, AUC, MCC, and clustering measures were not pooled. Even within PAM50 studies, sample selection, class count, and averaging differed. Accuracy could be driven by luminal A, while macro-F1 exposed weaker rare-class prediction. A multiclass AUC is uninterpretable without its one-versus-rest or one-versus-one construction and macro or micro averaging. Several papers reported gains over their own single-omic or concatenation baselines; those comparisons support a local contribution of the tested integration design but do not establish universal multi-omics superiority or a monotonic benefit from adding modalities.

### Interpretability and biological validation

Twenty-two of the 59 supervised studies used an explicit *post hoc* attribution or feature-importance method. SHAP predominated [28,31,33,41,46,48,50,62,64,77], GNNExplainer was used for hypergraph or graph models [49–51], integrated gradients was used in AMOGEL and GCOA-Net [47,63], and layer-wise relevance propagation or related relevance maps appeared in the attention-based GCN and METABRIC hybrid models [42,78]. Newer studies also used guided Grad-CAM, masking, permutation scores, and attention-derived feature rankings [55–57,59]. Twenty-five supervised studies reported pathway enrichment or another biological plausibility analysis, but only 2 examined selected biology in an external data resource. Only one explicitly tested explanation stability, fidelity, or robustness under the review criteria. These interpretability characteristics were reported separately from risk of bias.

These levels of evidence should not be conflated. A ranked feature list describes the model, enrichment tests the association of that list with annotated pathways, and external replication asks whether the association is reproduced elsewhere. None alone proves a causal mechanism. Explanations can vary across folds or correlated features even when predictive performance is stable. The limited evaluation of stability is therefore a major gap, especially for attention and graph methods marketed as interpretable.

## Discussion

### Principal findings and development of the field

Across the 2019 to 2026 publication window, the included studies show a clear technical shift. The earlier papers in this interval combined kernels, correlations, logistic evidence, forests, and constrained factors. Autoencoders then made modality-specific compression and joint latent learning common. Patient-similarity networks and GNNs shifted the computational object from a feature vector to relations among patients or genes. Hypergraphs introduced higher-order relations, while cross-omics attention enabled context-dependent exchange between modality embeddings. This change increased representational flexibility, but clinical evidence did not advance at the same rate. Only one complete model crossed cohorts, calibration appeared once, and no prospective or nested full-pipeline evaluation was identified.

The detailed study accounts also show that architecture labels hide substantial variation. Two “GNN” papers may use different node types, edge definitions, and fusion stages; 2 “autoencoder” papers may differ in whether encoders are pretrained, supervised, or variational; and 2 “transformers” may use self-attention within a modality or cross-attention between modalities. The orthogonal taxonomy is therefore more informative than a flat list of model families. It supports replication by specifying where fusion occurs, what is represented, how cross-modal information moves, what predicts the endpoint, and how the output is interpreted.

### What the studies collectively reveal about fusion

The dominance of intermediate fusion is understandable. It allows each omic to be normalized and encoded on its own scale and then combines smaller representations. This can prevent expression, with tens of thousands of variables, from overwhelming a lower-dimensional protein layer. It can also preserve source-specific encoders when one modality is noisier. The cost is a larger search space: encoder size, latent dimension, modality weights, and fusion operators all require tuning. Without nested or external evaluation, added flexibility may improve the fit to one cohort more than the underlying phenotype.

Kernel and similarity-network methods remain relevant because they integrate samples through comparable similarity matrices even when feature spaces differ. Factor and topic methods can yield compact, potentially interpretable programs. Graphs allow biological relationships or patient neighborhoods to shape prediction. Attention allows context-dependent weighting. None of these mechanisms is intrinsically superior. Their value depends on whether the imposed structure is scientifically defensible, learned without test-patient information, and stable across cohorts.

Late decision fusion was not represented as a sole primary design. This may reflect publication fashion rather than a negative result. Independent per-omic models can tolerate missing modalities and make the incremental value of each assay easier to inspect. A strong benchmark study should therefore compare early, intermediate, and late alternatives under the same patients, outer folds, parameter budget, and metrics, including missing-modality scenarios.

### Breast-cancer-specific constraints

PAM50 target dependence is the central interpretive constraint. Every 1 of the 57 intrinsic/PAM50 studies used expression, none reported excluding the PAM50 classifier genes or close proxies, and label provenance was clear in only 36. Predicting an expression-derived target from transcriptomics is therefore partly an assay-reconstruction problem. Thirty-nine studies included a matched expression-only baseline, and 35 reported a gain after adding at least one nonexpression layer, but these were usually internal comparisons and only one complete model crossed cohorts. Stronger studies could exclude the 50 classifier genes and close correlates from training, predict independently adjudicated immunohistochemistry-derived or clinical labels, and test the incremental value of nonexpression omics on a locked external cohort. Discovery studies should state whether PAM50 is used as a training signal, an evaluation reference, or only a descriptive annotation.

Class imbalance also requires breast-specific attention. Luminal A often dominates TCGA and METABRIC, whereas normal-like and HER2-enriched groups may be small. Overall accuracy can therefore look strong, while clinically important groups are poorly recognized. Macro-F1, balanced accuracy, macro-AUPRC, per-class recall, and confusion matrices should be prespecified. Resampling, synthetic oversampling, or class weighting must occur inside training folds. If it is applied before a split, nearby synthetic observations can carry information across the boundary.

Cohort reuse should not be described as independent confirmation. Sixty-two of the 70 publications used TCGA-BRCA and 9 used METABRIC, yet the corpus represented only 8 clearly named patient populations across all development, validation, and replication roles. TCGA-BRCA is invaluable but reflects particular inclusion criteria, tissue handling, and molecular platforms. METABRIC differs in platform and available layers, and clinical cohorts may have only 1 or 2 assays. Only Moanna evaluated the complete multi-omics model across named cohorts; other external analyses transferred a representation, a subtype signature, or selected biology rather than the locked full pipeline. A model requiring 5 complete omics can be impossible to transport even if its internal discrimination is high. Baena-Miret *et al.*’s block-wise missingness design [13] and cross-cohort evaluation by Lupat *et al.* [32] point toward the practical questions that need more attention.

Biological priors can help regularize small samples. Network filtering, pathway constraints, and knowledge graphs appeared in several studies [14,19,36,40,47,79]. In breast cancer, useful priors include ER signaling, HER2 amplification, proliferation and cell cycle programs, immune infiltration, and DNA repair processes. However, a prior can also reinforce existing annotation bias. Papers should report the version and provenance of the knowledge resource and compare against a matched model without the prior.

### Validation and reporting priorities

A credible multi-omics pipeline must split patients before any learned operation. Normalization parameters, imputation, feature selection, representation learning, patient-graph construction, and hyperparameter tuning should be fitted within the training data. Extensive tuning requires nested cross-validation or a locked external test set. For graph models, transductive use of test nodes should be declared and distinguished from inductive prediction for an unseen patient.

External validation should preserve the entire pipeline and document assay mapping, batch correction, unavailable features, missing modalities, and label harmonization. The external set should not be repeatedly consulted during development. When no cohort contains all required omics, authors can evaluate modality-dropout strategies, impute under realistic blocks, or prespecify a clinically deployable subset of assays.

Discrimination is not enough for a clinical prediction model. Calibration plots and a quantitative measure such as calibration slope, Brier score, or expected calibration error are needed to judge probabilities. CIs or repeated-resampling distributions should accompany principal metrics. Decision-curve analysis becomes meaningful only after a clinical decision and threshold are specified. Code should include preprocessing, splits, random seeds, and trained weights, not only a model class.

Interpretability should be treated as an evaluated model property. Feature attribution should be tested for fidelity to prediction, stability across folds and seeds, and robustness to modest perturbations. Enrichment analyses require a declared background universe and multiple-testing correction. External biological replication should use a dataset that did not influence model selection. Attention weights can guide exploration but do not automatically explain why a prediction changes.

### Relationship to adjacent breast cancer computational studies

Recent work in the *Computational and Structural Biotechnology Journal* illustrates the wider computational context: Raman classification of breast cancer cell lines, stain-free tissue imaging, mechanistic single-cell/multi-omics analysis, regional epithelial–mesenchymal transition expression, a UK Biobank subtype gap analysis, MRI response prediction, a multiscale molecular atlas, and cell-line genomic heterogeneity [80–87]. These studies contribute useful methods or biology, but they do not meet the operational requirement for a human subtype model that jointly uses at least 2 molecular omics. They are therefore contextual references rather than members of the 70-study set.

### Strengths and limitations

Strengths include database-specific strategies, exact exported-record counts, retained searches, explicit full-report exclusions, a documented inventory of 70 retrieved eligible studies, endpoint-stratified SWiM synthesis, a detailed account of what every study did, orthogonal coding of integration mechanisms, and separate assessment of risk of bias, reporting, and reproducibility; validation and clinical readiness; interpretability; discovery validity; PAM50 target dependence; and patient-population reuse. The search extended through 2026 September 8 and included 31 studies published in 2025 to 2026.

The limitations are material. Five of 82 sought reports remained unavailable for full-report assessment after publisher, institutional-access, bibliographic-index, Google Scholar, repository, and author-manuscript searches, with IEEE Xplore also checked for applicable IEEE-hosted reports. The smaller unresolved set reduces, but does not remove, the possibility of retrieval bias. Searches were restricted to English, 3 sources with reproducible exports, citation searching, and targeted supplementary searching. Google Scholar and IEEE Xplore did not generate independent bulk exports, so their incremental yield cannot be quantified separately, and the absence of separate Embase, Scopus, and Web of Science searches may have reduced sensitivity. The protocol was not prospectively registered. A second author completed a nonblinded check of the first author’s study-selection, extraction, and appraisal decisions; agreement was not quantified because this process did not produce 2 independent blinded rating sets. The appraisal was adapted to computational subtype studies and has not itself been externally validated. Incomplete reporting required conservative no/unclear coding and may underestimate procedures that authors performed but did not describe. Finally, most publications analyzed the same public cohorts, so the evidence base is methodologically diverse but not population diverse.

## Conclusions

AI-based multi-omics breast cancer subtyping has progressed from kernel and factor integration to modality-specific encoders, graphs, hypergraphs, and cross-omics attention. The included studies demonstrate many technically distinct ways to combine molecular information, but they remain dominated by PAM50 prediction in TCGA-BRCA. Architectural novelty and author-reported internal performance do not yet establish transportable clinical utility. The next stage of the field requires independent cohorts, fold-contained end-to-end analysis, endpoint and label clarity, class-sensitive metrics, calibrated uncertainty, missing-modality evaluation, reproducible code, and explanations tested for stability and biological replication.

## Ethical Approval

Not applicable; this study used published aggregate information and did not involve new participants or patient-level data collection.

## Data Availability

Report-level disposition, complete study-level extraction, taxonomy codes, audit judgments, and analysis counts are supplied in the Supplementary Materials and accompanying data files. Raw search exports and author-verification records are retained by the authors and are available from the corresponding author on reasonable request.

## Supplementary Materials

Supplementary 1 Supplementary Methods and Operational Definitions Methods Documentation Tables S1 to S8
