# Supplementary material for: Artificial Intelligence for Multi-omics Breast Cancer Subtype Prediction and Discovery: A Systematic Review
Source: Comput Struct Biotechnol J. 2026 Oct 26;2026issue-1(1):0243. doi: 10.34133/csbj.0243 (PMC13696802; doi:10.34133/csbj.0243)
Supplement: Supplementary file 1 [file csbj.0243.f1.docx]

**Supplementary Material
AI for Multi-Omics Breast Cancer Subtype Prediction and Discovery: A Systematic Review**
Deepali Pilodiya et al.
Electronic search date: 8 September 2026

# Supplementary methods and operational definitions

Eligibility required a human breast-cancer analysis that jointly used at least two molecular omics in one artificial-intelligence or machine-learning model for prespecified subtype prediction or explicit subtype discovery/recovery. Diagnosis-, stage-, prognosis-, survival-, recurrence-, metastasis- and treatment-response-only models were excluded. Imaging and clinical variables did not count as a second molecular omic. Synthesis was endpoint-stratified and narrative; performance estimates were neither pooled nor used to rank architectures. Prediction-specific characteristics were summarized among 59 supervised studies, discovery-specific characteristics among 11 discovery/recovery studies, and PAM50 target dependence among 57 intrinsic/PAM50 studies. Items outside a study’s endpoint were coded not applicable rather than absent.

# Methods documentation

The protocol was not prospectively registered. A written methods record was maintained during the review and covered eligibility criteria, information sources, record management, study selection, extraction fields, endpoint strata, the orthogonal integration taxonomy and the appraisal framework. It also documented the complete non-blinded second-author verification procedure, endpoint-applicable denominators, separate treatment of risk of bias and complementary evidence characteristics, the PAM50 target-dependence audit and the patient-population map. No part of this record is presented as a prospectively registered protocol.

# Table S1. Primary database searches and supplementary search routes

| **Source** | **Date** | **N** | **Search or retrieval procedure** |
| --- | --- | --- | --- |
| PubMed/MEDLINE | 8 September 2026 | 191 | (“breast neoplasms”[MeSH Terms] OR “breast cancer”[Title/Abstract] OR BRCA[Title/Abstract]) AND (“multi-omics”[Title/Abstract] OR multiomics[Title/Abstract] OR “multi omics”[Title/Abstract]) AND (subtype*[Title/Abstract] OR classif*[Title/Abstract] OR stratification[Title/Abstract]) AND (“machine learning”[Title/Abstract] OR “deep learning”[Title/Abstract] OR “artificial intelligence”[Title/Abstract] OR “neural network*”[Title/Abstract] OR transformer*[Title/Abstract] OR “graph neural network*”[Title/Abstract]) AND (“2019/01/01”[Date - Publication] : “2026/09/08”[Date - Publication]) AND english[Language] |
| Europe PMC | 8 September 2026 | 150 | (TITLE_ABS:“breast cancer” OR TITLE_ABS:BRCA) AND (TITLE_ABS:“multi-omics” OR TITLE_ABS:multiomics OR TITLE_ABS:“multi omics”) AND (TITLE_ABS:subtype* OR TITLE_ABS:classif* OR TITLE_ABS:stratification) AND (TITLE_ABS:“machine learning” OR TITLE_ABS:“deep learning” OR TITLE_ABS:“artificial intelligence” OR TITLE_ABS:“neural network*” OR TITLE_ABS:transformer* OR TITLE_ABS:“graph neural network*”) AND FIRST_PDATE:[2019-01-01 TO 2026-09-08] AND LANG:eng |
| OpenAlex API | 8 September 2026 | 364 | Four title-and-abstract searches: breast cancer multi-omics subtype classification (206); breast cancer multiomics subtype classification (59); BRCA multi-omics classification (86); BRCA multiomics classification (13). |
| Google Scholar | Supplementary | – | Targeted searches by exact title, author and method name were used to locate reports, publisher records, repositories and author manuscripts. This was not a bulk-export record stream; located citations were reconciled with the saved database and citation-search records. |
| IEEE Xplore | Supplementary | – | Title- and DOI-based lookup was used for relevant IEEE-hosted records and full texts identified through the primary sources, citation searching or Google Scholar. No independent bulk export was added to the 705-record database count. |

# Table S2. Record and report accounting

| **Step** | **N** | **Rule or disposition** |
| --- | --- | --- |
| Raw electronic records | 705 | 191 PubMed + 150 Europe PMC + 364 OpenAlex records. |
| Duplicates removed | 301 | Normalized DOI, then normalized title; author–year when DOI was absent. |
| Unique electronic records screened | 404 | All entered title/abstract screening; 333 were excluded. |
| Electronic reports sought / retrieved | 71 / 66 | Five reports remained unavailable after the documented retrieval attempts. |
| Electronic reports excluded / included | 5 / 61 | Full-report eligibility assessment. |
| Citation-search reports sought / retrieved | 11 / 11 | Backward and forward citation searching. |
| Citation reports excluded / included | 2 / 9 | Full-report eligibility assessment. |
| Total included studies | 70 | 61 electronic-route + 9 citation-route studies. |
| Supplementary routes | – | Google Scholar and IEEE Xplore supported targeted report location and retrieval; citations were reconciled with existing records and were not counted as a separate bulk export. |

# Table S3. Full-report exclusions

| **DOI** | **Title** | **Reason** |
| --- | --- | --- |
| [10.1007/s13369-024-09341-7](https://doi.org/10.1007/s13369-024-09341-7) | Multi-omics-based Machine Learning for the Subtype Classification of Breast Cancer | No integrated model: omics were analysed separately |
| [10.1177/11769351221124205](https://doi.org/10.1177/11769351221124205) | Multi-omics Data Integration Model Based on UMAP Embedding and Convolutional Neural Network. | Ineligible endpoint: tumour-stage classification |
| [10.1371/journal.pcbi.1014015](https://doi.org/10.1371/journal.pcbi.1014015) | CA-CAE: A deep learning-based multi-omics model for pan-cancer subtype classification and prognosis prediction. | No breast-cancer cohort or separately reported breast-cancer result |
| [10.3390/cancers18040593](https://doi.org/10.3390/cancers18040593) | Revisiting AI Interpretability in Precision Oncology: Why Predictive Accuracy Does Not Ensure Stable Feature Importance. | Ineligible endpoint: vital-status prediction |
| [10.1038/s41416-024-02587-w](https://doi.org/10.1038/s41416-024-02587-w) | wMKL: multi-omics data integration enables novel cancer subtype identification via weight-boosted multi-kernel learning. | No human breast-cancer model or separately reported breast-cancer result |
| [10.5626/jok.2020.47.9.835](https://doi.org/10.5626/jok.2020.47.9.835) | Breast Cancer Subtype Classification Using Multi-omics Data Integration Based on Neural Network | Non-English full report (Korean) |
| [10.1541/ieejeiss.146.321](https://doi.org/10.1541/ieejeiss.146.321) | Breast Cancer Subtype Classification via Multi-Omics Integration Using Bayesian Optimization and Contrastive Learning; | Non-English full report (Japanese) |

# Table S4. Reports not retrieved

These five reports were not counted as excluded because complete-report eligibility could not be established. They represent 5/82 (6.1%) reports sought. Twenty-one reports that were outstanding after the initial retrieval step were subsequently obtained and assessed.

| **DOI** | **Title** | **Route** | **Documented retrieval attempts** | **Eligibility signal before full report** |
| --- | --- | --- | --- | --- |
| [10.1021/acs.jcim.4c02130](https://doi.org/10.1021/acs.jcim.4c02130) | MOCapsNet: Multiomics Data Integration for Cancer Subtype Analysis Based on Dynamic Self-Attention Learning and Capsule Networks | Electronic databases | DOI and publisher landing page checked; available institutional-access route checked; Google Scholar, PubMed, Europe PMC and OpenAlex checked; exact-title/author searches and legitimate repository or author-manuscript routes checked. No usable full report was obtained. | Title/abstract indicated a multi-omics cancer-subtype model with a breast-cancer benchmark; full-report eligibility could not be confirmed. |
| [10.1080/03610918.2025.2550697](https://doi.org/10.1080/03610918.2025.2550697) | Subtype classification and lifetime prediction of cancer upon multi-omics permutation-GAT-GNNExplainer | Electronic databases | DOI and publisher landing page checked; available institutional-access route checked; Google Scholar, PubMed, Europe PMC and OpenAlex checked; exact-title/author searches and legitimate repository or author-manuscript routes checked. No usable full report was obtained. | Title/abstract indicated multi-omics subtype classification, but a separately extractable breast-cancer result could not be confirmed without the report. |
| [10.1109/tcbbio.2025.3613591](https://doi.org/10.1109/tcbbio.2025.3613591) | A Unified Genetic and Epigenetic Model to Predict Breast Cancer Intrinsic Subtypes Using Large DNA-Level Multi-Omics Data and Hierarchical Learning | Electronic databases | DOI and IEEE Xplore landing page checked; available institutional-access route checked; Google Scholar, PubMed, Europe PMC and OpenAlex checked; exact-title/author searches and legitimate repository or author-manuscript routes checked. No usable full report was obtained. | Abstract described DNA-level genetic and epigenetic intrinsic subtyping and therefore appeared potentially eligible. |
| [10.1177/09287329241296438](https://doi.org/10.1177/09287329241296438) | Deep learning-driven multi-omics sequential diagnosis with Hybrid-OmniSeq: Unraveling breast cancer complexity | Electronic databases | DOI and publisher landing page checked; available institutional-access route checked; Google Scholar, PubMed, Europe PMC and OpenAlex checked; exact-title/author searches and legitimate repository or author-manuscript routes checked. No usable full report was obtained. | Abstract described a METABRIC subtype analysis, but the actual molecular inputs and integrated model could not be verified from the abstract alone. |
| [10.2174/0115748936308171240605075531](https://doi.org/10.2174/0115748936308171240605075531) | Multinomial Logistic Regression with Adaptive Regularization for Cancer Subtype Classification via Multi-omics Data | Electronic databases | DOI and publisher landing page checked; available institutional-access route checked; Google Scholar, PubMed, Europe PMC and OpenAlex checked; exact-title/author searches and legitimate repository or author-manuscript routes checked. No usable full report was obtained. | Title/abstract indicated multi-omics cancer-subtype classification; breast-specific eligibility and methods required the full report. |

# Table S5A. Included-study design and data

| **Study** | **DOI** | **Endpoint** | **Class structure** | **Cohort** | **Breast-cancer sample size** | **Molecular omics** |
| --- | --- | --- | --- | --- | --- | --- |
| Tao M 2019 (2019)Classifying Breast Cancer Subtypes Using Multiple Kernel Learning Based on Omics Data. | [10.3390/genes10030200](https://doi.org/10.3390/genes10030200) | Intrinsic/PAM50 | Four- or five-class | TCGA-BRCA | 606 | mRNA/gene expression; DNA methylation; copy-number alteration |
| Xu J 2019 (2019)A hierarchical integration deep flexible neural forest framework for cancer subtype classification by integrating multi-omics data. | [10.1186/s12859-019-3116-7](https://doi.org/10.1186/s12859-019-3116-7) | Intrinsic/PAM50 | Four- or five-class | TCGA-BRCA | 104 BRCA complete cases | mRNA/gene expression; miRNA; DNA methylation |
| Chierici M 2020 (2020)Integrative Network Fusion: A Multi-Omics Approach in Molecular Profiling. | [10.3389/fonc.2020.01065](https://doi.org/10.3389/fonc.2020.01065) | Intrinsic/PAM50 | Four-class plus binary | TCGA-BRCA | 305 PAM50; 381 ER-status | mRNA/gene expression; miRNA; copy-number alteration; protein/RPPA |
| Lin Y 2020 (2020)Classifying Breast Cancer Subtypes Using Deep Neural Networks Based on Multi-Omics Data. | [10.3390/genes11080888](https://doi.org/10.3390/genes11080888) | Intrinsic/PAM50 | Four- or five-class | TCGA-BRCA | 606 | mRNA/gene expression; DNA methylation; copy-number alteration |
| Mohaiminul Islam M 2020 (2020)An integrative deep learning framework for classifying molecular subtypes of breast cancer. | [10.1016/j.csbj.2020.08.005](https://doi.org/10.1016/j.csbj.2020.08.005) | Intrinsic/PAM50 | Four- or five-class | METABRIC | 1,885 | mRNA/gene expression; DNA methylation; copy-number alteration; protein/RPPA |
| Zhang 2020 (2020)ELMO: An Efficient Logistic Regression-Based Multi-Omic Integrated Analysis Method for Breast Cancer Intrinsic Subtypes | [10.1109/access.2019.2960373](https://doi.org/10.1109/access.2019.2960373) | Intrinsic/PAM50 | Four- or five-class | TCGA-BRCA | 518 | mRNA/gene expression; miRNA; DNA methylation |
| El-Nabawy 2021 (2021)A Cascade Deep Forest Model for Breast Cancer Subtype Classification Using Multi-Omics Data | [10.3390/math9131574](https://doi.org/10.3390/math9131574) | Intrinsic/PAM50 | Four- or five-class | METABRIC | METABRIC discovery cohort; N not clearly stated | mRNA/gene expression; miRNA; copy-number alteration |
| Jung I 2021 (2021)MONTI: A Multi-Omics Non-negative Tensor Decomposition Framework for Gene-Level Integrative Analysis. | [10.3389/fgene.2021.682841](https://doi.org/10.3389/fgene.2021.682841) | Intrinsic/PAM50 | Four- or five-class | TCGA-BRCA | 597 | mRNA/gene expression; miRNA; protein/RPPA |
| Qi L 2021 (2021)Multi-Omics Data Fusion for Cancer Molecular Subtyping Using Sparse Canonical Correlation Analysis. | [10.3389/fgene.2021.607817](https://doi.org/10.3389/fgene.2021.607817) | Data-driven discovery/recovery | Data-driven | TCGA-BRCA; GSE22220 | 451 TCGA; 207 GSE22220 paired | mRNA/gene expression; miRNA; DNA methylation; copy-number alteration |
| Yang H 2021 (2021)Subtype-GAN: a deep learning approach for integrative cancer subtyping of multi-omics data | [10.1093/bioinformatics/btab109](https://doi.org/10.1093/bioinformatics/btab109) | Data-driven discovery/recovery | Five discovered clusters | TCGA-BRCA | 1031 | mRNA; miRNA; DNA methylation; copy-number alteration |
| Zhang L 2021 (2021)AutoGGN: A gene graph network AutoML tool for multi-omics research | [10.1016/j.ailsci.2021.100019](https://doi.org/10.1016/j.ailsci.2021.100019) | Intrinsic/PAM50 | Four-class | TCGA-BRCA | 396 | mRNA; protein/RPPA |
| Khan D 2022 (2022)Leveraging Deep Learning Techniques and Integrated Omics Data for Tailored Treatment of Breast Cancer. | [10.3390/jpm12050674](https://doi.org/10.3390/jpm12050674) | Intrinsic/PAM50 | Four- or five-class | TCGA-BRCA | 279 | mRNA/gene expression; miRNA; DNA methylation; copy-number alteration; somatic mutation; protein/RPPA |
| Li X 2022 (2022)MoGCN: A Multi-Omics Integration Method Based on Graph Convolutional Network for Cancer Subtype Analysis. | [10.3389/fgene.2022.806842](https://doi.org/10.3389/fgene.2022.806842) | Intrinsic/PAM50 | Four- or five-class | TCGA-BRCA | 511 | mRNA/gene expression; copy-number alteration; protein/RPPA |
| Meshoul 2022 (2022)Explainable Multi-Class Classification Based on Integrative Feature Selection for Breast Cancer Subtyping | [10.3390/math10224271](https://doi.org/10.3390/math10224271) | Intrinsic/PAM50 | Four- or five-class | TCGA-BRCA | 606 | mRNA/gene expression; miRNA; DNA methylation; copy-number alteration; protein/RPPA |
| Pouryahya M 2022 (2022)aWCluster: A Novel Integrative Network-Based Clustering of Multiomics for Subtype Analysis of Cancer Data. | [10.1109/tcbb.2020.3039511](https://doi.org/10.1109/tcbb.2020.3039511) | Data-driven discovery/recovery | Data-driven | TCGA-BRCA | 726 BRCA samples | mRNA/gene expression; DNA methylation; copy-number alteration |
| Valle F 2022 (2022)Multiomics Topic Modeling for Breast Cancer Classification. | [10.3390/cancers14051150](https://doi.org/10.3390/cancers14051150) | Data-driven discovery/recovery | Data-driven | TCGA-BRCA | TCGA-BRCA; N not clearly stated | mRNA/gene expression; miRNA; copy-number alteration |
| Viaud G 2022 (2022)Representation Learning for the Clustering of Multi-Omics Data | [10.1109/tcbb.2021.3060340](https://doi.org/10.1109/tcbb.2021.3060340) | Data-driven discovery/recovery | Two to nine candidate clusters | TCGA-BRCA | 348 | DNA methylation; miRNA; mRNA; protein/RPPA |
| Benkirane H 2023 (2023)CustOmics: A versatile deep-learning based strategy for multi-omics integration. | [10.1371/journal.pcbi.1010921](https://doi.org/10.1371/journal.pcbi.1010921) | Intrinsic/PAM50 | Four- or five-class | TCGA-BRCA | 1,022 | mRNA/gene expression; miRNA; DNA methylation; copy-number alteration; protein/RPPA |
| Choi JM 2023 (2023)moBRCA-net: a breast cancer subtype classification framework based on multi-omics attention neural networks. | [10.1186/s12859-023-05273-5](https://doi.org/10.1186/s12859-023-05273-5) | Intrinsic/PAM50 | Four- or five-class | TCGA-BRCA | 1,059 | mRNA/gene expression; miRNA; DNA methylation; copy-number alteration |
| Guo H 2023 (2023)Attention-based GCN integrates multi-omics data for breast cancer subtype classification and patient-specific gene marker identification | [10.1093/bfgp/elad013](https://doi.org/10.1093/bfgp/elad013) | Intrinsic/PAM50 | Multiclass | TCGA-BRCA | Not clearly stated in the report | mRNA; DNA methylation; copy-number alteration |
| Kesimoglu ZN 2023 (2023)SUPREME: multiomics data integration using graph convolutional networks. | [10.1093/nargab/lqad063](https://doi.org/10.1093/nargab/lqad063) | Intrinsic/PAM50 | Four- or five-class | TCGA-BRCA; METABRIC; combined TCGA+METABRIC | 1,022 TCGA; 1,699 METABRIC; 2,721 combined | mRNA/gene expression; miRNA; DNA methylation; copy-number alteration |
| Lupat 2023 (2023)Moanna: Multi-Omics Autoencoder-Based Neural Network Algorithm for Predicting Breast Cancer Subtypes | [10.1109/access.2023.3240515](https://doi.org/10.1109/access.2023.3240515) | Intrinsic/PAM50 | Four- or five-class | METABRIC development; TCGA-BRCA external test | 1,689 METABRIC; 954 TCGA | mRNA/gene expression; copy-number alteration; somatic mutation |
| Unlu Yazici M 2023 (2023)Invention of 3Mint for feature grouping and scoring in multi-omics. | [10.3389/fgene.2023.1093326](https://doi.org/10.3389/fgene.2023.1093326) | Intrinsic/PAM50 | Four- or five-class | TCGA-BRCA | 372 (248 luminal; 124 ER-negative) | mRNA/gene expression; miRNA; DNA methylation; copy-number alteration; protein/RPPA |
| Cheng L 2024 (2024)MoAGL-SA: a multi-omics adaptive integration method with graph learning and self attention for cancer subtype classification. | [10.1186/s12859-024-05989-y](https://doi.org/10.1186/s12859-024-05989-y) | Intrinsic/PAM50 | Four- or five-class | TCGA-BRCA | Not clearly reported | mRNA/gene expression; miRNA; DNA methylation |
| Gygi JP 2024 (2024)A supervised Bayesian factor model for the identification of multi-omics signatures. | [10.1093/bioinformatics/btae202](https://doi.org/10.1093/bioinformatics/btae202) | Intrinsic/PAM50 | Four- or five-class | TCGA-BRCA | 989 | mRNA/gene expression; miRNA |
| Huang Y 2024 (2024)Classifying breast cancer subtypes on multi-omics data via sparse canonical correlation analysis and deep learning. | [10.1186/s12859-024-05749-y](https://doi.org/10.1186/s12859-024-05749-y) | Intrinsic/PAM50 | Four- or five-class | TCGA-BRCA | 528 complete cases | mRNA/gene expression; DNA methylation |
| Li B 2024 (2024)A multimodal graph neural network framework for cancer molecular subtype classification. | [10.1186/s12859-023-05622-4](https://doi.org/10.1186/s12859-023-05622-4) | Intrinsic/PAM50 | Four- or five-class | TCGA-BRCA | 981 | mRNA/gene expression; miRNA; DNA methylation; copy-number alteration |
| Liu Z 2024 (2024)DMOIT: denoised multi-omics integration approach based on transformer multi-head self-attention mechanism. | [10.3389/fgene.2024.1488683](https://doi.org/10.3389/fgene.2024.1488683) | Intrinsic/PAM50 | Four- or five-class | TCGA-BRCA | TCGA-BRCA; N not clearly stated | mRNA/gene expression; DNA methylation; copy-number alteration; protein/RPPA |
| Ren Y 2024 (2024)Classifying breast cancer using multi-view graph neural network based on multi-omics data. | [10.3389/fgene.2024.1363896](https://doi.org/10.3389/fgene.2024.1363896) | Intrinsic/PAM50 | Four- or five-class | TCGA-BRCA | 606 | mRNA/gene expression; DNA methylation; copy-number alteration; protein/RPPA |
| Shen J 2024 (2024)CAEM-GBDT: a cancer subtype identifying method using multi-omics data and convolutional autoencoder network. | [10.3389/fbinf.2024.1403826](https://doi.org/10.3389/fbinf.2024.1403826) | Intrinsic/PAM50 | Four- or five-class | Public breast-cancer cohort (name not clear) | 104 BRCA complete cases | mRNA/gene expression; miRNA; DNA methylation; protein/RPPA |
| Tang X 2024 (2024)OmicsFootPrint: a framework to integrate and interpret multi-omics data using circular images and deep neural networks. | [10.1093/nar/gkae915](https://doi.org/10.1093/nar/gkae915) | Intrinsic/PAM50 | Four- or five-class | TCGA-BRCA | TCGA-BRCA; N not clearly stated for PAM50 task | mRNA/gene expression; miRNA; copy-number alteration; somatic mutation; protein/RPPA |
| Tanvir 2024 (2024)MOGAT: A Multi-Omics Integration Framework Using Graph Attention Networks for Cancer Subtype Prediction | [10.3390/ijms25052788](https://doi.org/10.3390/ijms25052788) | Intrinsic/PAM50 | Four- or five-class | TCGA-BRCA | 920 | mRNA/gene expression; miRNA; DNA methylation; copy-number alteration |
| Wang H 2024 (2024)HyperTMO: a trusted multi-omics integration framework based on hypergraph convolutional network for patient classification | [10.1093/bioinformatics/btae159](https://doi.org/10.1093/bioinformatics/btae159) | Intrinsic/PAM50 | Five-class | TCGA-BRCA | Not stated for BRCA in the main report | mRNA; DNA methylation; miRNA |
| Wang J 2024 (2024)A semi-supervised approach for the integration of multi-omics data based on transformer multi-head self-attention mechanism and graph convolutional networks. | [10.1186/s12864-024-09985-7](https://doi.org/10.1186/s12864-024-09985-7) | Intrinsic/PAM50 | Four- or five-class | Public breast-cancer cohort (name not clear) | TCGA-BRCA; N not clearly stated | mRNA/gene expression; miRNA; DNA methylation; copy-number alteration; protein/RPPA |
| Wu J 2024 (2024)DeepMoIC: multi-omics data integration via deep graph convolutional networks for cancer subtype classification. | [10.1186/s12864-024-11112-5](https://doi.org/10.1186/s12864-024-11112-5) | Intrinsic/PAM50 | Four- or five-class | TCGA-BRCA | 511 | mRNA/gene expression; miRNA; DNA methylation; copy-number alteration; protein/RPPA |
| Yang S 2024 (2024)Comparative Evaluation of Machine Learning Models for Subtyping Triple-Negative Breast Cancer: A Deep Learning-Based Multi-Omics Data Integration Approach. | [10.7150/jca.93215](https://doi.org/10.7150/jca.93215) | TNBC status | Binary | TCGA-BRCA; TCIA imaging subset | Up to 1,097 per omic; matched-model N not clearly reported | mRNA/gene expression; miRNA; DNA methylation; copy-number alteration; somatic mutation |
| Yao 2024 (2024)MOCAT: multi-omics integration with auxiliary classifiers enhanced autoencoder | [10.1186/s13040-024-00360-6](https://doi.org/10.1186/s13040-024-00360-6) | Intrinsic/PAM50 | Four- or five-class | Public breast-cancer cohort (name not clear) | 875 | mRNA/gene expression; miRNA; protein/RPPA |
| Zeng 2024 (2024)DiffRS-net: A Novel Framework for Classifying Breast Cancer Subtypes on Multi-Omics Data | [10.3390/app14072728](https://doi.org/10.3390/app14072728) | Intrinsic/PAM50 | Four- or five-class | TCGA-BRCA | 520 | mRNA/gene expression; miRNA; DNA methylation |
| Zhang 2024 (2024)AET-net: A framework for subtype classification based on the multi-omics data of breast cancer | [10.62617/mcb785](https://doi.org/10.62617/mcb785) | Intrinsic/PAM50 | Four- or five-class | TCGA-BRCA | 780 before SMOTE | mRNA/gene expression; DNA methylation; protein/RPPA |
| Baena-Miret 2025 (2025)A Two-Step Algorithm for Handling Block-Wise Missing Data in Multi-Omics | [10.3390/app15073650](https://doi.org/10.3390/app15073650) | Intrinsic/PAM50 | Four- or five-class | TCGA-BRCA | 528 | mRNA/gene expression; protein/RPPA |
| Bong F 2025 (2025)Augmented kurtosis-based projection pursuit: a novel, advanced machine learning approach for multi-omics data analysis and integration. | [10.1093/nar/gkaf844](https://doi.org/10.1093/nar/gkaf844) | Data-driven discovery/recovery | Data-driven | TCGA-BRCA; AURORA-US (biological replication) | 1,057 TCGA; 129 AURORA transcriptomes | mRNA/gene expression; DNA methylation; protein/RPPA |
| Hu GM 2025 (2025)Mapping cancer heterogeneity: a consensus network approach to subtypes and pathways. | [10.1093/bib/bbaf452](https://doi.org/10.1093/bib/bbaf452) | Data-driven discovery/recovery | Data-driven | TCGA-BRCA | TCGA-BRCA; N not clearly stated | mRNA/gene expression; miRNA; copy-number alteration; protein/RPPA |
| Muneeba Afzal Mukhdoomi 2025 (2025)PHYPERNET: P-LAPLACIAN REGULARIZATION HYPERGRAPH LEARNING FOR MULTI-OMICS CANCER SUBTYPING | [10.12732/ijam.v38i2s.71](https://doi.org/10.12732/ijam.v38i2s.71) | Data-driven discovery/recovery | Data-driven | TCGA-BRCA | TCGA-BRCA; N not clearly stated | mRNA/gene expression; miRNA; DNA methylation; copy-number alteration; protein/RPPA |
| Omran MM 2025 (2025)Comparative analysis of statistical and deep learning-based multi-omics integration for breast cancer subtype classification. | [10.1186/s12967-025-06662-5](https://doi.org/10.1186/s12967-025-06662-5) | Intrinsic/PAM50 | Four- or five-class | TCGA-BRCA | 960 | mRNA/gene expression; miRNA |
| Patel JC 2025 (2025)GAIN-BRCA: a graph-based AI-net framework for breast cancer subtype classification using multiomics data. | [10.1093/bioadv/vbaf116](https://doi.org/10.1093/bioadv/vbaf116) | Intrinsic/PAM50 | Four- or five-class | TCGA-BRCA | 669 | mRNA/gene expression; miRNA; DNA methylation; copy-number alteration; protein/RPPA |
| Pateras J 2025 (2025)Heterogeneous Clustering of Multiomics Data for Breast Cancer Subgroup Classification and Detection. | [10.3390/ijms26041707](https://doi.org/10.3390/ijms26041707) | Data-driven discovery/recovery | Data-driven | TCGA-BRCA | 302 | mRNA/gene expression; miRNA; DNA methylation; copy-number alteration; protein/RPPA |
| Tan CY 2025 (2025)Amogel: a multi-omics classification framework using associative graph neural networks with prior knowledge for biomarker identification. | [10.1186/s12859-025-06111-6](https://doi.org/10.1186/s12859-025-06111-6) | Intrinsic/PAM50 | Four- or five-class | TCGA-BRCA | 985 | mRNA/gene expression; miRNA; DNA methylation; protein/RPPA |
| Tan K 2025 (2025)GL-Fusion: A Multi-Omics Integration Method Based on Graph-Level Structure Fusion and Locus-Level Feature Fusion for Cancer Subtype Classification | [10.1109/jbhi.2025.3624554](https://doi.org/10.1109/jbhi.2025.3624554) | Intrinsic/PAM50 | Five-class | TCGA-BRCA | 770 | mRNA; DNA methylation; somatic copy-number variation |
| Unlu Yazici M 2025 (2025)3Mont: A multi-omics integrative tool for breast cancer subtype stratification. | [10.1371/journal.pone.0326154](https://doi.org/10.1371/journal.pone.0326154) | Receptor status | Binary | TCGA-BRCA | 549 (425 HR+; 124 HR-) | mRNA/gene expression; miRNA; DNA methylation; protein/RPPA |
| Zhang G 2025 (2025)MOFNet: a deep learning framework for multi-omics data fusion in cancer subtype classification | [10.1039/d5mo00221d](https://doi.org/10.1039/d5mo00221d) | Intrinsic/PAM50 | Five-class | TCGA-BRCA | 875 | mRNA; DNA methylation; miRNA |
| Zhao G 2025 (2025)TreeEM: Tree-enhanced ensemble model combining with feature selection for cancer subtype classification and survival prediction | [10.1016/j.rinam.2025.100605](https://doi.org/10.1016/j.rinam.2025.100605) | Intrinsic/PAM50 | Pairwise binary tasks among four classes | TCGA-BRCA | 511 | copy-number alteration; mRNA; protein/RPPA |
| Atfa 2026 (2026)COAT: A Cross-Omics Attention Transformer for PAM50 Breast Cancer Subtype Classification | [10.14569/ijacsa.2026.0170537](https://doi.org/10.14569/ijacsa.2026.0170537) | Intrinsic/PAM50 | Four- or five-class | TCGA-BRCA | 824 | mRNA/gene expression; miRNA; DNA methylation |
| Fu X 2026 (2026)FastMI-HGNet: A Two-Stream Heterogeneous Graph Neural Network for Multi-Omics Disease Classification. | [10.3390/genes17080865](https://doi.org/10.3390/genes17080865) | Intrinsic/PAM50 | Four- or five-class | TCGA-BRCA | BRCA N not stated in main text | mRNA/gene expression; miRNA; DNA methylation; protein/RPPA |
| Guo M 2026 (2026)Robust graph structure learning to improve multi-omics cancer subtype classification. | [10.1186/s12859-026-06404-4](https://doi.org/10.1186/s12859-026-06404-4) | Intrinsic/PAM50 | Four- or five-class | TCGA-BRCA | 450 PAM50 samples | mRNA/gene expression; miRNA; copy-number alteration; somatic mutation; protein/RPPA |
| Huang Y 2026 (2026)UMCA-Net: Uncertainty-aware multi-stage cross-attention for cost-aware multi-omics data classification | [10.1016/j.compbiomed.2026.111860](https://doi.org/10.1016/j.compbiomed.2026.111860) | Intrinsic/PAM50 | Five-class | TCGA-BRCA | 876 | mRNA; DNA methylation; miRNA |
| Lan G 2026 (2026)PIK3CA Pan-Cancer Analysis Highlights Its Critical Role in BRCA and Development of TCGA-Derived GNN Model for PAM50 Subtype Prediction | [10.1155/ijcp/6988098](https://doi.org/10.1155/ijcp/6988098) | Intrinsic/PAM50 | Five-class | TCGA-BRCA | Not clearly stated for the matched BRCA analysis | mRNA; DNA methylation; miRNA |
| Li C 2026 (2026)GCOA-Net: a graph-regularized cross-omics attention network for interpretable breast cancer molecular subtype classification. | [10.3389/fmed.2026.1790437](https://doi.org/10.3389/fmed.2026.1790437) | Intrinsic/PAM50 | Four- or five-class | TCGA-BRCA | 1,059 | mRNA/gene expression; miRNA; DNA methylation |
| Li M 2026 (2026)Multiview-cooperated graph neural network enables novel multi-omics cancer subtype classification | [10.1016/j.compbiolchem.2025.108665](https://doi.org/10.1016/j.compbiolchem.2025.108665) | Intrinsic/PAM50 | Five-class | TCGA-BRCA | 875 | mRNA; DNA methylation; miRNA |
| Li Q 2026 (2026)MoACNN-XGNet: Interpretable Multi-Omics Convolutional Network for Breast Cancer Subtyping and Prognostic Genes Identification | [10.1109/jbhi.2025.3595381](https://doi.org/10.1109/jbhi.2025.3595381) | Intrinsic/PAM50 | Four-class | TCGA-BRCA; METABRIC; GSE84207 | 721 TCGA development cases; 1157 METABRIC cases | copy-number alteration; mRNA; DNA methylation |
| Meng P 2026 (2026)MOTCS: A Cancer Subtype Classification and Key Biomarker Recognition Model Based on Multi-Omics Data Integration of Transformer. | [10.3390/ijms27104237](https://doi.org/10.3390/ijms27104237) | Intrinsic/PAM50 | Four- or five-class | TCGA-BRCA | BRCA subset of 2,033 across six cancers; BRCA N not stated | mRNA/gene expression; miRNA; DNA methylation; protein/RPPA |
| Mu N 2026 (2026)An uncertainty-aware dynamic decision framework for progressive multi-omics integration in classification tasks | [10.1016/j.cmpb.2025.109179](https://doi.org/10.1016/j.cmpb.2025.109179) | Intrinsic/PAM50 | Five-class | TCGA-BRCA | 875-876 (reported inconsistently by table/description) | mRNA; DNA methylation; miRNA |
| Siam MBH 2026 (2026)Effects of similarity networks in graph-based multi-omics classification. | [10.1371/journal.pone.0344754](https://doi.org/10.1371/journal.pone.0344754) | Intrinsic/PAM50 | Four- or five-class | Public breast-cancer cohort (name not clear) | TCGA-BRCA; N not clearly stated | mRNA/gene expression; miRNA; DNA methylation; protein/RPPA |
| Subramaniam M 2026 (2026)Integration of multi-omics data accelerates via credal set interval neural networks for breast cancer molecular subtype classification with high resolution predictive analytics | [10.1016/j.bspc.2025.109298](https://doi.org/10.1016/j.bspc.2025.109298) | Intrinsic/PAM50 | Five-class | TCGA-BRCA | 528 matched cases stated; confusion-matrix totals are not internally consistent | mRNA; DNA methylation |
| Wang J 2026 (2026)Subtype-HM: A Novel Cancer Subtype Identification Method Based on Hypergraph Learning and Multi-omics Data | [10.1007/s12539-025-00789-1](https://doi.org/10.1007/s12539-025-00789-1) | Data-driven discovery/recovery | Five discovered clusters | TCGA-BRCA | 1031 | mRNA; miRNA; DNA methylation; copy-number alteration |
| Wang T 2026 (2026)Integrative multi-omics clustering for identifying novel breast cancer subtypes with distinct molecular and clinical characteristics | [10.1016/j.intonc.2025.12.001](https://doi.org/10.1016/j.intonc.2025.12.001) | Data-driven discovery/recovery | Five discovered clusters | TCGA-BRCA; GSE202203; GSE96058; METABRIC; NKI | 670 TCGA-BRCA discovery cases | mRNA; miRNA; lncRNA; DNA methylation; copy-number alteration; somatic mutation |
| Wu H 2026 (2026)MOGANet: A Multi-omics Graph Attention Network for Cancer Diagnosis and Biomarker Identification | [10.1007/s12539-026-00828-5](https://doi.org/10.1007/s12539-026-00828-5) | Intrinsic/PAM50 | Five-class | TCGA-BRCA | Approximately 875 | mRNA; DNA methylation; miRNA |
| Yang Y 2026 (2026)MuLeCoG: multi-level contrastive graph network for cancer subtype classification | [10.1080/10255842.2026.2642810](https://doi.org/10.1080/10255842.2026.2642810) | Intrinsic/PAM50 | Four-class | TCGA-BRCA | 646 | mRNA; DNA methylation; miRNA |
| Zhang G 2026 (2026)AttentionCCA: An attention-based canonical correlation analysis framework for integrative multi-omics cancer stratification | [10.1016/j.cmpb.2026.109562](https://doi.org/10.1016/j.cmpb.2026.109562) | Intrinsic/PAM50 | Four-class | TCGA-BRCA; METABRIC expression-only transfer | 430 development; 1826 expression-only external cases | mRNA; DNA methylation; miRNA |
| Zhumagozhayev 2026 (2026)Analysis of Multi-Omics Data in Breast Cancer Subtype Classification | [10.1109/access.2026.3695279](https://doi.org/10.1109/access.2026.3695279) | Intrinsic/PAM50 | Four- or five-class | METABRIC | 1,523 | mRNA/gene expression; miRNA; DNA methylation; copy-number alteration; somatic mutation; protein/RPPA |
| Zhumagozhayev 2026 (2026)Cross-Modal Self-Attention Fusion for Breast Cancer Subtype Classification Using Multi-Omics Data | [10.23950/jcmk/18568](https://doi.org/10.23950/jcmk/18568) | Intrinsic/PAM50 | Four- or five-class | METABRIC | 1,523 | mRNA/gene expression; miRNA; DNA methylation; copy-number alteration; somatic mutation |

# Table S5B. Integration, modelling, validation and reported results

Results are retained as author-reported study characteristics. Heterogeneous metrics, patient sets, label definitions and validation designs precluded pooling or architecture ranking.

| **Study** | **Fusion** | **Representation** | **Interaction** | **Predictor** | **Attention** | **Validation** | **Author-reported result summary** |
| --- | --- | --- | --- | --- | --- | --- | --- |
| Tao M 2019 (2019) | Early or hybrid; see report | Tabular/kernel | Kernel combination/correlation | Kernel/correlation model | None/unclear | 10-fold cross-validation | No single comparable summary value extracted; reported metrics: accuracy; ROC-AUC; precision/recall. |
| Xu J 2019 (2019) | Intermediate (learned representation fusion) | Tabular/kernel | Concatenation/joint encoding | Tree/forest ensemble | None/unclear | 5-fold cross-validation | No single comparable summary value extracted; reported metrics: accuracy; precision/recall. |
| Chierici M 2020 (2020) | Hybrid early/intermediate (juxtaposition plus SNF) | Tabular/kernel | Concatenation/joint encoding | Machine-learning classifier | None/unclear | 5-fold cross-validation | No single comparable summary value extracted; reported metrics: accuracy; MCC; precision/recall; clustering/biological association. |
| Lin Y 2020 (2020) | Intermediate (graph-based representation fusion) | Learned latent representation | Concatenation/joint encoding | Autoencoder/deep neural network | None/unclear | 5-fold cross-validation | No single comparable summary value extracted; reported metrics: accuracy; ROC-AUC; precision/recall. |
| Mohaiminul Islam M 2020 (2020) | Intermediate (learned representation fusion) | Learned latent representation | Concatenation/joint encoding | Autoencoder/deep neural network | None/unclear | 10-fold cross-validation | No single comparable summary value extracted; reported metrics: accuracy; ROC-AUC; precision/recall; clustering/biological association. |
| Zhang 2020 (2020) | Intermediate (logistic evidence integrated across omics) | Tabular/kernel | Decision/evidence aggregation | Linear/regularized machine learning | None/unclear | 10-fold cross-validation | No single comparable summary value extracted; reported metrics: accuracy; ROC-AUC; precision/recall; clustering/biological association. |
| El-Nabawy 2021 (2021) | Intermediate (learned representation fusion) | Tabular/kernel | Concatenation/joint encoding | Tree/forest ensemble | None/unclear | Internal validation; details not clearly reported | No single comparable summary value extracted; reported metrics: accuracy; precision/recall. |
| Jung I 2021 (2021) | Intermediate (graph-based representation fusion) | Tensor | Joint factorization/decomposition | Probabilistic/factor/clustering model | None/unclear | 10-fold cross validation | No single comparable summary value extracted; reported metrics: accuracy; macro/weighted F1. |
| Qi L 2021 (2021) | Early or hybrid; see report | Tabular/kernel | Kernel combination/correlation | Kernel/correlation model | None/unclear | Internal validation; details not clearly reported | No single comparable summary value extracted; reported metrics: accuracy; precision/recall; clustering/biological association. |
| Yang H 2021 (2021) | Intermediate latent fusion | Adversarial autoencoder latent representation | Multiple-input/multiple-output adversarial learning followed by consensus Gaussian-mixture clustering | Adversarial autoencoder plus Gaussian-mixture clustering | None reported | Consensus subsampling, survival and clinical association analyses | The model selected five BRCA clusters and related them to survival and clinical characteristics |
| Zhang L 2021 (2021) | Hybrid graph and feature fusion | Gene/PPI graph plus modality-specific arrays | GCN message passing followed by CNN-based fusion | Graph convolutional network and convolutional classifier | None reported | 10% hold-out test; repeated five-fold cross-validation on the remaining data | Held-out accuracy 86.3% for the four-class breast-cancer task |
| Khan D 2022 (2022) | Intermediate (learned representation fusion) | Learned latent representation | Concatenation/joint encoding | Autoencoder/deep neural network | None/unclear | five-fold cross-validation | No single comparable summary value extracted; reported metrics: accuracy; ROC-AUC; precision/recall; clustering/biological association. |
| Li X 2022 (2022) | Intermediate (graph-based representation fusion) | Sample/gene graph | Message passing/similarity propagation | Graph neural network | None/unclear | 10-fold cross validation | No single comparable summary value extracted; reported metrics: accuracy; macro/weighted F1; precision/recall; clustering/biological association. |
| Meshoul 2022 (2022) | Early or hybrid; see report | Tabular/kernel | Concatenation/joint encoding | Linear/regularized machine learning | None/unclear | Internal validation; details not clearly reported | No single comparable summary value extracted; reported metrics: accuracy; ROC-AUC. |
| Pouryahya M 2022 (2022) | Intermediate (aggregated omics distance networks) | Sample/gene graph | Message passing/similarity propagation | Probabilistic/factor/clustering model | None/unclear | Internal validation; details not clearly reported | No single comparable summary value extracted; reported metrics: accuracy; clustering/biological association. |
| Valle F 2022 (2022) | Intermediate (multipartite topic model) | Topic model | Joint factorization/decomposition | Probabilistic/factor/clustering model | None/unclear | Internal validation; details not clearly reported | No single comparable summary value extracted; reported metrics: accuracy; precision/recall; clustering/biological association. |
| Viaud G 2022 (2022) | Intermediate latent fusion | PCA/MFA factors and stacked or disjoint autoencoder embeddings | Joint and source-balanced latent representation followed by k-means | k-means on statistical or autoencoder representations | – | Internal-index, cluster-number and architecture/source-balance sensitivity analyses | Compared statistical and deep representations and showed that architecture and source balance changed the retained clustering structure |
| Benkirane H 2023 (2023) | Intermediate (learned representation fusion) | Learned latent representation | Concatenation/joint encoding | Autoencoder/deep neural network | None/unclear | 5-fold cross-validation | No single comparable summary value extracted; reported metrics: accuracy; macro/weighted F1; ROC-AUC; precision/recall; clustering/biological association. |
| Choi JM 2023 (2023) | Intermediate (modality encoders with omics attention) | Learned latent representation | Self/graph attention | Transformer/attention neural network | Self/graph attention | 10-fold cross validation | No single comparable summary value extracted; reported metrics: accuracy; macro/weighted F1; MCC; clustering/biological association. |
| Guo H 2023 (2023) | Intermediate graph fusion | PPI-informed gene graph | Graph convolution with row-wise, column-wise and squeeze-and-excitation attention | Attention-based graph convolutional network | Row-wise and column-wise self-attention; squeeze-and-excitation | Five-fold cross-validation | Column-attention AGCN reported AUC 0.9816, accuracy 0.8743 and MCC 0.8151 in five-fold cross-validation |
| Kesimoglu ZN 2023 (2023) | Intermediate/late (network-specific embeddings plus raw features) | Sample/gene graph | Message passing/similarity propagation | Graph neural network | None/unclear | Internal train/validation/test or hold-out split | No single comparable summary value extracted; reported metrics: accuracy; macro/weighted F1; precision/recall; clustering/biological association. |
| Lupat 2023 (2023) | Intermediate (joint autoencoder representation) | Learned latent representation | Concatenation/joint encoding | Autoencoder/deep neural network | None/unclear | Hold-out METABRIC plus independent TCGA test | No single comparable summary value extracted; reported metrics: accuracy; macro/weighted F1; precision/recall; clustering/biological association. |
| Unlu Yazici M 2023 (2023) | Early or hybrid; see report | Tabular/kernel | Concatenation/joint encoding | Linear/regularized machine learning | None/unclear | Internal train/validation/test or hold-out split | Authors reported 0.9235 accuracy and 0.9212 F1 for the principal comparison. |
| Cheng L 2024 (2024) | Intermediate (graph-based representation fusion) | Sample/gene graph | Self/graph attention | Transformer/attention neural network | Self/graph attention | Internal validation; details not clearly reported | Authors reported 88.22% accuracy and 83.83% macro-F1 for BRCA. |
| Gygi JP 2024 (2024) | Intermediate (joint supervised latent factors) | Learned latent representation | Joint factorization/decomposition | Probabilistic/factor/clustering model | None/unclear | Internal train/validation/test or hold-out split | No single comparable summary value extracted; reported metrics: accuracy; ROC-AUC. |
| Huang Y 2024 (2024) | Intermediate (learned representation fusion) | Tabular/kernel | Kernel combination/correlation | Kernel/correlation model | None/unclear | Internal validation; details not clearly reported | No single comparable summary value extracted; reported metrics: accuracy; macro/weighted F1; ROC-AUC; precision/recall. |
| Li B 2024 (2024) | Intermediate (graph-based representation fusion) | Sample/gene graph | Message passing/similarity propagation | Graph neural network | None/unclear | Internal validation; details not clearly reported | No single comparable summary value extracted; reported metrics: accuracy; macro/weighted F1; precision/recall. |
| Liu Z 2024 (2024) | Intermediate (learned representation fusion) | Learned latent representation | Self/graph attention | Transformer/attention neural network | Self/graph attention | 5-fold cross-validation | No single comparable summary value extracted; reported metrics: accuracy; macro/weighted F1. |
| Ren Y 2024 (2024) | Intermediate (graph-based representation fusion) | Sample/gene graph | Message passing/similarity propagation | Graph neural network | None/unclear | 5-fold cross-validation | No single comparable summary value extracted; reported metrics: accuracy; macro/weighted F1; ROC-AUC; precision/recall. |
| Shen J 2024 (2024) | Intermediate (graph-based representation fusion) | Learned latent representation | Concatenation/joint encoding | Tree/forest ensemble | None/unclear | five-fold cross-validation | No single comparable summary value extracted; reported metrics: accuracy; macro/weighted F1; precision/recall. |
| Tang X 2024 (2024) | Intermediate (graph-based representation fusion) | Image-transformed/tabular | Concatenation/joint encoding | Autoencoder/deep neural network | None/unclear | 10-fold cross-validation | No single comparable summary value extracted; reported metrics: accuracy; ROC-AUC; clustering/biological association. |
| Tanvir 2024 (2024) | Intermediate (graph-based representation fusion) | Sample/gene graph | Self/graph attention | Transformer/attention neural network | Self/graph attention | Internal validation; details not clearly reported | Authors reported macro-F1 of 0.804 for BRCA. |
| Wang H 2024 (2024) | Late evidential fusion | Omic-specific patient hypergraphs | Hypergraph convolution followed by Dempster-Shafer evidence aggregation | Hypergraph convolutional networks | None reported | Five-fold cross-validation | BRCA accuracy 0.858 +/- 0.023, weighted F1 0.863 +/- 0.023 and macro-F1 0.841 +/- 0.019 |
| Wang J 2024 (2024) | Intermediate (graph-based representation fusion) | Sample/gene graph | Self/graph attention | Transformer/attention neural network | Self/graph attention | Internal validation; details not clearly reported | No single comparable summary value extracted; reported metrics: accuracy; macro/weighted F1; ROC-AUC; precision/recall. |
| Wu J 2024 (2024) | Intermediate (graph-based representation fusion) | Sample/gene graph | Message passing/similarity propagation | Graph neural network | None/unclear | Internal validation; details not clearly reported | Authors reported 92.98% accuracy and 91.52% F1 for BRCA. |
| Yang S 2024 (2024) | Early (feature-level molecular fusion) | Learned latent representation | Concatenation/joint encoding | Autoencoder/deep neural network | None/unclear | 10-fold cross-validation | No single comparable summary value extracted; reported metrics: accuracy; macro/weighted F1; ROC-AUC; precision/recall. |
| Yao 2024 (2024) | Intermediate (learned representation fusion) | Learned latent representation | Concatenation/joint encoding | Autoencoder/deep neural network | None/unclear | Internal validation; details not clearly reported | No single comparable summary value extracted; reported metrics: accuracy; macro/weighted F1; ROC-AUC; precision/recall. |
| Zeng 2024 (2024) | Early or hybrid; see report | Tabular/kernel | Concatenation/joint encoding | Machine-learning classifier | None/unclear | 5-fold cross-validation | No single comparable summary value extracted; reported metrics: accuracy; precision/recall. |
| Zhang 2024 (2024) | Early or hybrid; see report | Tabular/kernel | Concatenation/joint encoding | Machine-learning classifier | None/unclear | Internal validation; details not clearly reported | No single comparable summary value extracted; reported metrics: accuracy; macro/weighted F1; ROC-AUC; precision/recall. |
| Baena-Miret 2025 (2025) | Early or hybrid; see report | Tabular/kernel | Concatenation/joint encoding | Machine-learning classifier | None/unclear | Internal validation; details not clearly reported | No single comparable summary value extracted; reported metrics: accuracy; precision/recall. |
| Bong F 2025 (2025) | Early or hybrid; see report | Tabular/kernel | Concatenation/joint encoding | Probabilistic/factor/clustering model | None/unclear | Internal validation; details not clearly reported | No single comparable summary value extracted; reported metrics: precision/recall; clustering/biological association. |
| Hu GM 2025 (2025) | Intermediate (graph-based representation fusion) | Tabular/kernel | Concatenation/joint encoding | Probabilistic/factor/clustering model | None/unclear | Internal validation; details not clearly reported | No single comparable summary value extracted; reported metrics: accuracy; ROC-AUC; precision/recall; clustering/biological association. |
| Muneeba Afzal Mukhdoomi 2025 (2025) | Intermediate/late (hypergraph evidence or embedding fusion) | Hypergraph | Message passing/similarity propagation | Hypergraph neural network | None/unclear | Internal validation; details not clearly reported | No single comparable summary value extracted; reported metrics: accuracy; macro/weighted F1; precision/recall; clustering/biological association. |
| Omran MM 2025 (2025) | Intermediate (learned representation fusion) | Learned latent representation | Concatenation/joint encoding | Autoencoder/deep neural network | None/unclear | five-fold cross-validation | Best reported F1 was 0.75 for the nonlinear model using MOFA+ features. |
| Patel JC 2025 (2025) | Early (omics-associated graph before learning) | Sample/gene graph | Message passing/similarity propagation | Graph neural network | None/unclear | five-fold cross-validation | No single comparable summary value extracted; reported metrics: accuracy; macro/weighted F1; ROC-AUC; precision/recall; clustering/biological association. |
| Pateras J 2025 (2025) | Early or hybrid; see report | Tabular/kernel | Joint factorization/decomposition | Probabilistic/factor/clustering model | None/unclear | Internal validation; details not clearly reported | No single comparable summary value extracted; reported metrics: accuracy; precision/recall; clustering/biological association. |
| Tan CY 2025 (2025) | Intermediate (graph-based representation fusion) | Sample/gene graph | Message passing/similarity propagation | Graph neural network | None/unclear | Internal train/validation/test or hold-out split | Authors reported mean BRCA accuracy of 76.6% (SD 2.4). |
| Tan K 2025 (2025) | Graph-level and locus-level hybrid fusion | Gene graphs and locus-aligned features | Similarity-network graph fusion, structural entropy optimization and locus-level graph convolution | Graph convolutional network | None reported | Five-fold cross-validation | BRCA accuracy 90.65% (SD 2.27%); omics-combination experiments showed RNA as the strongest single layer and the full model as the strongest reported combination |
| Unlu Yazici M 2025 (2025) | Early (feature groups across omics) | Tabular/kernel | Concatenation/joint encoding | Linear/regularized machine learning | None/unclear | Internal train/validation/test or hold-out split | No single comparable summary value extracted; reported metrics: accuracy; ROC-AUC; precision/recall; clustering/biological association. |
| Zhang G 2025 (2025) | Intermediate plus label-space fusion | Omic-specific similarity graphs | Graph pooling and graph convolution followed by VCDN label-space fusion | Graph convolutional network plus view-correlation discovery network | Graph pooling weights | Repeated stratified 70/30 splits | BRCA accuracy 0.8517 +/- 0.015, weighted F1 0.8536 +/- 0.017 and macro-F1 0.8093 +/- 0.024 |
| Zhao G 2025 (2025) | Early feature fusion | Tabular features | Concatenation and tree-ensemble voting | Undersampling random forest and extremely randomized trees | – | Five-fold cross-validation | Reported pairwise subtype results; no single directly comparable multiclass estimate |
| Atfa 2026 (2026) | Intermediate (modality encoders plus cross-attention) | Learned latent representation | Cross-attention | Transformer/attention neural network | Cross-attention | 10-FOLD CROSS-VALIDATION | No single comparable summary value extracted; reported metrics: accuracy; macro/weighted F1; ROC-AUC; precision/recall. |
| Fu X 2026 (2026) | Intermediate (graph-based representation fusion) | Sample/gene graph | Message passing/similarity propagation | Graph neural network | None/unclear | 5-fold cross-validation | No single comparable summary value extracted; reported metrics: accuracy; ROC-AUC; precision/recall. |
| Guo M 2026 (2026) | Intermediate (graph-based representation fusion) | Sample/gene graph | Message passing/similarity propagation | Graph neural network | None/unclear | five-fold cross-validation | No single comparable summary value extracted; reported metrics: accuracy; macro/weighted F1; precision/recall; clustering/biological association. |
| Huang Y 2026 (2026) | Progressive cross-attention fusion | Modality-specific latent tokens | Multi-stage cross-attention with evidential uncertainty and cost-aware modality acquisition | Transformer-style evidential network | Cross-attention | Internal splits over five random seeds; exact partition details insufficiently clear | BRCA mRNA-only accuracy 0.8670 and tri-omics accuracy 0.8652; the full model did not improve the matched expression baseline |
| Lan G 2026 (2026) | Intermediate plus decision fusion | Omic-specific patient graphs | Per-omic graph convolution followed by VCDN or mean fusion | Graph convolutional networks | None reported | Five random stratified 70/30 splits | VCDN accuracy 0.8213, macro-F1 0.7877 and weighted F1 0.8283; mean fusion accuracy 0.8555, macro-F1 0.8211 and weighted F1 0.8599 |
| Li C 2026 (2026) | Intermediate (graph-based representation fusion) | Sample/gene graph | Cross-attention | Transformer/attention neural network | Cross-attention | Repeated stratified cross-validation | No single comparable summary value extracted; reported metrics: accuracy; macro/weighted F1; ROC-AUC; AUPRC; MCC; precision/recall. |
| Li M 2026 (2026) | Intermediate plus label-space fusion | Mahalanobis/density patient graphs | Stacked graph convolution, inter-view attention and VCDN | Multiview graph neural network | Inter-view attention | Five-fold cross-validation | BRCA accuracy 0.791 +/- 0.021, weighted F1 0.791 +/- 0.008 and balanced accuracy 0.742 +/- 0.012 |
| Li Q 2026 (2026) | Early image-level fusion | Two-dimensional image-transformed omics | Convolution and attention across image-transformed modalities | Attention-based convolutional neural network | Channel/spatial attention | Five-fold cross-validation and 7:2:1 hold-out; METABRIC same-method analysis and GSE84207 methylation-only biological replication | TCGA full-model accuracy 91.67%; single-omic accuracies were 66.43% (CNV), 78.09% (methylation) and 79.86% (expression); METABRIC same-method accuracy 92.45% |
| Meng P 2026 (2026) | Intermediate (learned representation fusion) | Learned latent representation | Self/graph attention | Transformer/attention neural network | Self/graph attention | 5-fold cross-validation | No single comparable summary value extracted; reported metrics: accuracy; macro/weighted F1; precision/recall. |
| Mu N 2026 (2026) | Progressive late evidential fusion | Modality-specific latent evidence vectors | Dirichlet evidential learning and Dempster-Shafer aggregation with on-demand modality acquisition | Evidential neural networks | Dynamic modality selection | Internal benchmark split; exact split procedure not sufficiently clear | Full BRCA model reported accuracy 0.855, F1 0.858 and macro-F1 0.829; the mRNA-only accuracy was higher (0.866) |
| Siam MBH 2026 (2026) | Intermediate (graph-based representation fusion) | Sample/gene graph | Message passing/similarity propagation | Graph neural network | None/unclear | Internal validation; details not clearly reported | No single comparable summary value extracted; reported metrics: accuracy; macro/weighted F1; ROC-AUC; precision/recall; clustering/biological association. |
| Subramaniam M 2026 (2026) | Intermediate latent fusion | Disentangled graph variational latent representation and credal intervals | Latent graph fusion with interval-valued credal decision layer | Credal set interval neural network optimized by chaotic Puma algorithm | None reported | 70/15/15 split with five-fold cross-validation described in training | Very high internal performance was reported, but sample totals in the confusion matrices do not reconcile with the stated cohort size |
| Wang J 2026 (2026) | Intermediate hypergraph integration | Multi-level hypergraphs | Hypergraph propagation, discriminator-guided attention and contrastive entropy alignment | Hypergraph clustering network | Discriminator-guided omic-specific attention | Ten rounds of 80% subsampling, parameter sensitivity, survival, clinical and enrichment analyses | Five BRCA subtypes were analysed; repeated subsampling and sensitivity analyses evaluated cluster robustness |
| Wang T 2026 (2026) | Consensus across intermediate integration methods | Consensus of ten multi-omics clustering representations | MOVICS consensus integration | Consensus clustering and expression-based nearest-template/classifier transfer | – | Gap/CPI/silhouette, leave-one-omics-out concordance and four independent expression cohorts | Five TCGA subtypes were selected; leave-one-omics-out concordance exceeded 85%, and expression signatures were examined in four independent cohorts |
| Wu H 2026 (2026) | Intermediate hierarchical attention fusion | Modality-specific patient graphs | Per-view GCNs followed by hierarchical attention-based fusion | Graph convolutional network | Intra-view and hierarchical inter-view attention | Repeated 70/30 splits; five-fold cross-validation for hyperparameter selection in training data | MOGANet outperformed the reported BRCA benchmark comparators; exact metrics are retained in Supplementary Table S5B |
| Yang Y 2026 (2026) | Intermediate multi-view graph fusion | Modality-specific patient graphs | Hierarchical intra- and inter-view contrastive learning followed by representation fusion | Graph representation network with SVM classifier | Graph-level weighting | Ten repetitions of stratified five-fold cross-validation; SVM tuning inside training data | Reported mean BRCA accuracy around 0.84-0.86 across the principal and ablation tables |
| Zhang G 2026 (2026) | Intermediate correlation and attention fusion | Sparse canonical variates | Weighted CCA, feature attention and cross-modal attention | AttentionCCA representation plus classifier | Feature and cross-modal attention | Ten-fold cross-validation; METABRIC expression-only transfer analysis | TCGA-BRCA accuracy 0.9261 and macro-F1 0.9162; the full multi-omics macro score exceeded the matched mRNA-only result |
| Zhumagozhayev 2026 (2026) | Early or hybrid; see report | Tabular/kernel | Concatenation/joint encoding | Machine-learning classifier | None/unclear | 5-fold cross-validation | No single comparable summary value extracted; reported metrics: accuracy; macro/weighted F1; ROC-AUC; precision/recall. |
| Zhumagozhayev 2026 (2026) | Intermediate (modality encoders plus cross-attention) | Learned latent representation | Self/graph attention | Transformer/attention neural network | Self/graph attention | 5-fold cross-validation | No single comparable summary value extracted; reported metrics: accuracy; macro/weighted F1; ROC-AUC; precision/recall. |

# Table S5C. Preprocessing, feature selection, reproducibility and interpretation

| **Study** | **Preprocessing** | **Feature selection** | **Class imbalance** | **Code/software** | **Interpretation and biological checks** |
| --- | --- | --- | --- | --- | --- |
| Tao M 2019 (2019) | normalization/scaling; missing-data filtering/imputation | regularization/embedded selection; pathway/network filtering | No/unclear | No/unclear | None reported; biological plausibility: No/unclear; external biological replication: No |
| Xu J 2019 (2019) | normalization/scaling; missing-data filtering/imputation | regularization/embedded selection; representation learning | No/unclear | No/unclear | None reported; biological plausibility: No/unclear; external biological replication: No |
| Chierici M 2020 (2020) | normalization/scaling; log transformation | univariate/statistical filtering; regularization/embedded selection | No/unclear | Yes | None reported; biological plausibility: Yes; external biological replication: No |
| Lin Y 2020 (2020) | normalization/scaling; missing-data filtering/imputation | univariate/statistical filtering; regularization/embedded selection | Yes | Yes | None reported; biological plausibility: No/unclear; external biological replication: No |
| Mohaiminul Islam M 2020 (2020) | normalization/scaling | univariate/statistical filtering; regularization/embedded selection | Yes | No/unclear | None reported; biological plausibility: No/unclear; external biological replication: No |
| Zhang 2020 (2020) | normalization/scaling; missing-data filtering/imputation | regularization/embedded selection | No/unclear | No/unclear | None reported; biological plausibility: No/unclear; external biological replication: No |
| El-Nabawy 2021 (2021) | missing-data filtering/imputation | regularization/embedded selection; representation learning | Yes | No/unclear | None reported; biological plausibility: No/unclear; external biological replication: No |
| Jung I 2021 (2021) | normalization/scaling; missing-data filtering/imputation | regularization/embedded selection; pathway/network filtering | No/unclear | Yes | None reported; biological plausibility: No/unclear; external biological replication: No |
| Qi L 2021 (2021) | normalization/scaling | regularization/embedded selection; pathway/network filtering | No/unclear | No/unclear | None reported; biological plausibility: No/unclear; external biological replication: No |
| Yang H 2021 (2021) | Modality-specific scaling and multiple-input processing | Adversarial representation learning; no separate supervised feature-selection step | No/unclear | Yes | None reported; biological plausibility: Yes; external biological replication: No |
| Zhang L 2021 (2021) | Gene-level expression and RPPA features aligned to a protein-interaction graph; normal samples excluded | AutoML searched graph and convolutional components; detailed fold containment unclear | No/unclear | Yes (AutoGGN software site) | SHAP; biological plausibility: Yes; external biological replication: No |
| Khan D 2022 (2022) | normalization/scaling; log transformation; missing-data filtering/imputation | regularization/embedded selection; representation learning | Yes | Yes | None reported; biological plausibility: No/unclear; external biological replication: No |
| Li X 2022 (2022) | normalization/scaling | representation learning; pathway/network filtering | No/unclear | Yes | None reported; biological plausibility: Yes; external biological replication: No |
| Meshoul 2022 (2022) | normalization/scaling; missing-data filtering/imputation | univariate/statistical filtering; regularization/embedded selection | No/unclear | Yes | SHAP; biological plausibility: No/unclear; external biological replication: No |
| Pouryahya M 2022 (2022) | normalization/scaling | pathway/network filtering | No/unclear | Yes | None reported; biological plausibility: Yes; external biological replication: No |
| Valle F 2022 (2022) | normalization/scaling; missing-data filtering/imputation | pathway/network filtering | No/unclear | Yes | None reported; biological plausibility: No/unclear; external biological replication: No |
| Viaud G 2022 (2022) | Source-wise transformations appropriate to PCA/MFA and autoencoders | Representation learning rather than explicit feature selection | No/unclear | No/unclear | None reported; biological plausibility: No/unclear; external biological replication: No |
| Benkirane H 2023 (2023) | normalization/scaling; log transformation; missing-data filtering/imputation | regularization/embedded selection; representation learning | No/unclear | Yes | SHAP; biological plausibility: Yes; external biological replication: No |
| Choi JM 2023 (2023) | normalization/scaling; log transformation; missing-data filtering/imputation | univariate/statistical filtering; regularization/embedded selection | No/unclear | Yes | None reported; biological plausibility: No/unclear; external biological replication: No |
| Guo H 2023 (2023) | Gene-level modalities mapped to a protein-protein interaction graph | Graph-based representation; fold containment of all preprocessing unclear | No/unclear | Yes (GCN_multiomics repository) | Layer-wise relevance propagation; biological plausibility: Yes; external biological replication: No |
| Kesimoglu ZN 2023 (2023) | normalization/scaling | representation learning; pathway/network filtering | No/unclear | Yes | None reported; biological plausibility: No/unclear; external biological replication: No |
| Lupat 2023 (2023) | normalization/scaling; batch correction | univariate/statistical filtering; representation learning | No/unclear | Yes | None reported; biological plausibility: No/unclear; external biological replication: No |
| Unlu Yazici M 2023 (2023) | normalization/scaling; log transformation; missing-data filtering/imputation | univariate/statistical filtering; pathway/network filtering | No/unclear | Yes | None reported; biological plausibility: Yes; external biological replication: No |
| Cheng L 2024 (2024) | Not clearly reported | representation learning | No/unclear | No/unclear | None reported; biological plausibility: Yes; external biological replication: No |
| Gygi JP 2024 (2024) | normalization/scaling | regularization/embedded selection; representation learning | No/unclear | Yes | None reported; biological plausibility: No/unclear; external biological replication: No |
| Huang Y 2024 (2024) | normalization/scaling | univariate/statistical filtering; regularization/embedded selection | No/unclear | Yes | None reported; biological plausibility: No/unclear; external biological replication: No |
| Li B 2024 (2024) | normalization/scaling | regularization/embedded selection; pathway/network filtering | No/unclear | No/unclear | None reported; biological plausibility: No/unclear; external biological replication: No |
| Liu Z 2024 (2024) | normalization/scaling; missing-data filtering/imputation | univariate/statistical filtering; regularization/embedded selection | No/unclear | No/unclear | None reported; biological plausibility: No/unclear; external biological replication: No |
| Ren Y 2024 (2024) | normalization/scaling | univariate/statistical filtering; regularization/embedded selection | No/unclear | No/unclear | None reported; biological plausibility: No/unclear; external biological replication: No |
| Shen J 2024 (2024) | normalization/scaling | regularization/embedded selection; representation learning | No/unclear | Yes | None reported; biological plausibility: No/unclear; external biological replication: No |
| Tang X 2024 (2024) | normalization/scaling | univariate/statistical filtering; regularization/embedded selection | No/unclear | Yes | SHAP; biological plausibility: Yes; external biological replication: No |
| Tanvir 2024 (2024) | Not clearly reported | regularization/embedded selection; representation learning | No/unclear | Yes | None reported; biological plausibility: No/unclear; external biological replication: No |
| Wang H 2024 (2024) | MOGONET-preprocessed features used to construct one hypergraph per omic | Hypergraph representation and wrapper-based biomarker ranking | No/unclear | Yes | Feature-importance/ablation ranking; biological plausibility: Yes; external biological replication: No |
| Wang J 2024 (2024) | normalization/scaling | univariate/statistical filtering; regularization/embedded selection | No/unclear | No/unclear | None reported; biological plausibility: No/unclear; external biological replication: No |
| Wu J 2024 (2024) | normalization/scaling; missing-data filtering/imputation; batch correction | representation learning; pathway/network filtering | No/unclear | Yes | None reported; biological plausibility: Yes; external biological replication: No |
| Yang S 2024 (2024) | normalization/scaling; missing-data filtering/imputation | univariate/statistical filtering; regularization/embedded selection | No/unclear | No/unclear | None reported; biological plausibility: Yes; external biological replication: No |
| Yao 2024 (2024) | normalization/scaling | regularization/embedded selection; representation learning | No/unclear | Yes | None reported; biological plausibility: No/unclear; external biological replication: No |
| Zeng 2024 (2024) | normalization/scaling; missing-data filtering/imputation | univariate/statistical filtering | No/unclear | Yes | None reported; biological plausibility: No/unclear; external biological replication: No |
| Zhang 2024 (2024) | normalization/scaling | representation learning; pathway/network filtering | Yes | No/unclear | None reported; biological plausibility: No/unclear; external biological replication: No |
| Baena-Miret 2025 (2025) | missing-data filtering/imputation | regularization/embedded selection | No/unclear | Yes | None reported; biological plausibility: No/unclear; external biological replication: No |
| Bong F 2025 (2025) | normalization/scaling; missing-data filtering/imputation | univariate/statistical filtering; regularization/embedded selection | No/unclear | Yes | None reported; biological plausibility: No/unclear; external biological replication: Yes |
| Hu GM 2025 (2025) | normalization/scaling; log transformation; batch correction | pathway/network filtering | No/unclear | No/unclear | None reported; biological plausibility: Yes; external biological replication: No |
| Muneeba Afzal Mukhdoomi 2025 (2025) | Not clearly reported | regularization/embedded selection; representation learning | No/unclear | No/unclear | GNNExplainer; biological plausibility: Yes; external biological replication: No |
| Omran MM 2025 (2025) | normalization/scaling; batch correction | regularization/embedded selection; representation learning | No/unclear | No/unclear | SHAP; biological plausibility: Yes; external biological replication: No |
| Patel JC 2025 (2025) | normalization/scaling; missing-data filtering/imputation | univariate/statistical filtering; regularization/embedded selection | Yes | Yes | SHAP; biological plausibility: No/unclear; external biological replication: No |
| Pateras J 2025 (2025) | Not clearly reported | univariate/statistical filtering; regularization/embedded selection | No/unclear | No/unclear | None reported; biological plausibility: Yes; external biological replication: No |
| Tan CY 2025 (2025) | normalization/scaling; missing-data filtering/imputation | univariate/statistical filtering; regularization/embedded selection | No/unclear | Yes | Integrated Gradients; biological plausibility: Yes; external biological replication: Yes |
| Tan K 2025 (2025) | Differential filtering followed by graph construction; containment of filtering within folds unclear | Differential analysis and graph-entropy optimization | No/unclear | Yes | None reported; biological plausibility: Yes; external biological replication: No |
| Unlu Yazici M 2025 (2025) | normalization/scaling; missing-data filtering/imputation | univariate/statistical filtering; regularization/embedded selection | Yes | Yes | None reported; biological plausibility: Yes; external biological replication: No |
| Zhang G 2025 (2025) | Training-only feature selection and omic-specific graph construction | Similarity graph pooling retained 25% of features; fitted within training data | Yes (inverse-frequency loss) | Yes | Graph feature-importance scores; biological plausibility: Yes; external biological replication: No |
| Zhao G 2025 (2025) | Matched samples with high-dimensional omic features | Minimum-redundancy maximum-relevance; dimension selected using cross-validation without a reported nested outer loop | Yes (undersampling ensemble) | No/unclear | None reported; biological plausibility: No/unclear; external biological replication: No |
| Atfa 2026 (2026) | normalization/scaling | univariate/statistical filtering; regularization/embedded selection | Yes | No/unclear | SHAP; biological plausibility: Yes; external biological replication: No |
| Fu X 2026 (2026) | normalization/scaling; missing-data filtering/imputation; batch correction | univariate/statistical filtering; representation learning | No/unclear | No/unclear | SHAP; biological plausibility: Yes; external biological replication: No |
| Guo M 2026 (2026) | normalization/scaling; missing-data filtering/imputation | univariate/statistical filtering; regularization/embedded selection | No/unclear | Yes | GNNExplainer; biological plausibility: No/unclear; external biological replication: No |
| Huang Y 2026 (2026) | Modality-specific encoders on benchmark features | Representation learning; full fold containment unclear | No/unclear | Yes | None reported; biological plausibility: No/unclear; external biological replication: No |
| Lan G 2026 (2026) | Matched TCGA omics with modality-specific graph construction | Preselected benchmark features; containment unclear | No/unclear | No/unclear | None reported; biological plausibility: No/unclear; external biological replication: No |
| Li C 2026 (2026) | normalization/scaling | regularization/embedded selection; representation learning | No/unclear | No/unclear | Integrated Gradients; biological plausibility: No/unclear; external biological replication: No |
| Li M 2026 (2026) | Benchmark features with modality-specific patient graphs | Fixed benchmark feature dimensions; full fold containment unclear | No/unclear | No/unclear | Masking-based feature importance; biological plausibility: Yes; external biological replication: No |
| Li Q 2026 (2026) | UMAP converted all samples to two-dimensional omic images before cross-validation | UMAP plus convolutional feature learning | No/unclear | No/unclear | Guided Grad-CAM; biological plausibility: Yes; external biological replication: Yes |
| Meng P 2026 (2026) | normalization/scaling | regularization/embedded selection; representation learning | No/unclear | Yes | SHAP; biological plausibility: Yes; external biological replication: No |
| Mu N 2026 (2026) | Modality-specific neural encoders on standard benchmark features | Benchmark feature sets; fold containment not fully described | No/unclear | Yes | None reported; biological plausibility: No/unclear; external biological replication: No |
| Siam MBH 2026 (2026) | normalization/scaling; batch correction | regularization/embedded selection; representation learning | No/unclear | No/unclear | SHAP; GNNExplainer; biological plausibility: No/unclear; external biological replication: No |
| Subramaniam M 2026 (2026) | Kriged marginalized particle filtering and graph-variational representation learning | Model-based feature construction; fold containment unclear | No/unclear | No/unclear | None reported; biological plausibility: No/unclear; external biological replication: No |
| Wang J 2026 (2026) | Omic-specific feature sets used to construct multi-level hypergraphs | Hypergraph representation learning without a separate supervised selector | No/unclear | Yes | None reported; biological plausibility: Yes; external biological replication: No |
| Wang T 2026 (2026) | Six matched TCGA omics processed in the MOVICS workflow | Multi-algorithm consensus clustering and subtype-specific marker selection | No/unclear | Yes | None reported; biological plausibility: Yes; external biological replication: Yes |
| Wu H 2026 (2026) | Training-only scaling and feature selection stated | Fold-contained modality-specific filtering | No/unclear | Yes | Attention/feature-importance scores; biological plausibility: Yes; external biological replication: No |
| Yang Y 2026 (2026) | ANOVA feature selection performed separately within each cross-validation training fold | Fold-contained ANOVA filtering | Yes (balanced class weights) | No/unclear | Permutation feature importance; biological plausibility: Yes; external biological replication: No |
| Zhang G 2026 (2026) | Modality-wise standardization and sparse CCA feature learning | Weighted sparse canonical-correlation loadings with feature attention | No/unclear | Yes | Attention-derived feature importance; biological plausibility: Yes; external biological replication: No |
| Zhumagozhayev 2026 (2026) | Not clearly reported | univariate/statistical filtering; regularization/embedded selection | No/unclear | No/unclear | LRP; biological plausibility: Yes; external biological replication: No |
| Zhumagozhayev 2026 (2026) | normalization/scaling; log transformation; missing-data filtering/imputation | univariate/statistical filtering; regularization/embedded selection | Yes | No/unclear | None reported; biological plausibility: No/unclear; external biological replication: No |

# Table S6A. Risk of bias in supervised prediction studies (n=59)

The four domain judgements were adapted from PROBAST+AI. Overall risk of bias was derived only from these domains; reporting, reproducibility, interpretability and clinical-readiness items did not determine the overall judgement.

| **Study** | **Participants/data** | **Predictors/preprocessing** | **Outcome/reference standard** | **Analysis** | **Overall** |
| --- | --- | --- | --- | --- | --- |
| Tao M 2019 (2019) | Some concerns | High | Some concerns (expression-derived target) | High | High |
| Xu J 2019 (2019) | Some concerns | High | Some concerns (expression-derived target) | High | High |
| Chierici M 2020 (2020) | Some concerns | Some concerns | Some concerns (expression-derived target) | Some concerns | Some concerns |
| Lin Y 2020 (2020) | Some concerns | High | Some concerns (expression-derived target) | High | High |
| Mohaiminul Islam M 2020 (2020) | Some concerns | High | Some concerns (expression-derived target) | High | High |
| Zhang 2020 (2020) | Some concerns | High | Some concerns (expression-derived target) | High | High |
| El-Nabawy 2021 (2021) | Some concerns | High | Some concerns (expression-derived target) | High | High |
| Jung I 2021 (2021) | Some concerns | Some concerns | Some concerns (expression-derived target) | Some concerns | Some concerns |
| Zhang L 2021 (2021) | Some concerns | High | Some concerns (expression-derived target) | Some concerns | High |
| Khan D 2022 (2022) | Some concerns | High | Some concerns (expression-derived target) | High | High |
| Li X 2022 (2022) | Some concerns | High | Some concerns (expression-derived target) | High | High |
| Meshoul 2022 (2022) | Some concerns | High | Some concerns (expression-derived target) | High | High |
| Benkirane H 2023 (2023) | Some concerns | High | Some concerns (expression-derived target) | High | High |
| Choi JM 2023 (2023) | Some concerns | Some concerns | Some concerns (expression-derived target) | Some concerns | Some concerns |
| Guo H 2023 (2023) | Some concerns | High | Some concerns (expression-derived target) | High | High |
| Kesimoglu ZN 2023 (2023) | Some concerns | High | Some concerns (expression-derived target) | Some concerns | High |
| Lupat 2023 (2023) | Some concerns | High | Some concerns (expression-derived target) | Low | High |
| Unlu Yazici M 2023 (2023) | Some concerns | High | Some concerns (expression-derived target) | High | High |
| Cheng L 2024 (2024) | Some concerns | High | Some concerns (expression-derived target) | High | High |
| Gygi JP 2024 (2024) | Some concerns | High | Some concerns (expression-derived target) | Some concerns | High |
| Huang Y 2024 (2024) | Some concerns | High | Some concerns (expression-derived target) | High | High |
| Li B 2024 (2024) | Some concerns | High | Some concerns (expression-derived target) | High | High |
| Liu Z 2024 (2024) | Some concerns | High | Some concerns (expression-derived target) | High | High |
| Ren Y 2024 (2024) | Some concerns | High | Some concerns (expression-derived target) | High | High |
| Shen J 2024 (2024) | Some concerns | High | Some concerns (expression-derived target) | High | High |
| Tang X 2024 (2024) | Some concerns | High | Some concerns (expression-derived target) | High | High |
| Tanvir 2024 (2024) | Some concerns | High | Some concerns (expression-derived target) | High | High |
| Wang H 2024 (2024) | Some concerns | High | Some concerns (expression-derived target) | High | High |
| Wang J 2024 (2024) | Some concerns | High | Some concerns (expression-derived target) | High | High |
| Wu J 2024 (2024) | Some concerns | High | Some concerns (expression-derived target) | High | High |
| Yang S 2024 (2024) | Some concerns | High | Low | High | High |
| Yao 2024 (2024) | Some concerns | High | Some concerns (expression-derived target) | High | High |
| Zeng 2024 (2024) | Some concerns | High | Some concerns (expression-derived target) | High | High |
| Zhang 2024 (2024) | Some concerns | High | Some concerns (expression-derived target) | High | High |
| Baena-Miret 2025 (2025) | Some concerns | High | Some concerns (expression-derived target) | High | High |
| Omran MM 2025 (2025) | Some concerns | High | Some concerns (expression-derived target) | High | High |
| Patel JC 2025 (2025) | Some concerns | High | Some concerns (expression-derived target) | High | High |
| Tan CY 2025 (2025) | Some concerns | Some concerns | Some concerns (expression-derived target) | Some concerns | Some concerns |
| Tan K 2025 (2025) | Some concerns | High | Some concerns (expression-derived target) | High | High |
| Unlu Yazici M 2025 (2025) | Some concerns | High | Low | Some concerns | High |
| Zhang G 2025 (2025) | Some concerns | Some concerns | Some concerns (expression-derived target) | Some concerns | Some concerns |
| Zhao G 2025 (2025) | Some concerns | High | Some concerns (expression-derived target) | High | High |
| Atfa 2026 (2026) | Some concerns | Some concerns | Some concerns (expression-derived target) | Some concerns | Some concerns |
| Fu X 2026 (2026) | Some concerns | Some concerns | Some concerns (expression-derived target) | Some concerns | Some concerns |
| Guo M 2026 (2026) | Some concerns | High | Some concerns (expression-derived target) | High | High |
| Huang Y 2026 (2026) | Some concerns | High | Some concerns (expression-derived target) | High | High |
| Lan G 2026 (2026) | Some concerns | High | Some concerns (expression-derived target) | High | High |
| Li C 2026 (2026) | Some concerns | Some concerns | Some concerns (expression-derived target) | Some concerns | Some concerns |
| Li M 2026 (2026) | Some concerns | High | Some concerns (expression-derived target) | High | High |
| Li Q 2026 (2026) | Some concerns | High | Some concerns (expression-derived target) | High | High |
| Meng P 2026 (2026) | Some concerns | High | Some concerns (expression-derived target) | High | High |
| Mu N 2026 (2026) | Some concerns | High | Some concerns (expression-derived target) | High | High |
| Siam MBH 2026 (2026) | Some concerns | High | Some concerns (expression-derived target) | High | High |
| Subramaniam M 2026 (2026) | High | High | Some concerns (expression-derived target) | High | High |
| Wu H 2026 (2026) | Some concerns | Some concerns | Some concerns (expression-derived target) | Some concerns | Some concerns |
| Yang Y 2026 (2026) | Some concerns | Some concerns | Some concerns (expression-derived target) | Some concerns | Some concerns |
| Zhang G 2026 (2026) | Some concerns | High | Some concerns (expression-derived target) | High | High |
| Zhumagozhayev 2026 (2026) | Some concerns | High | Some concerns (expression-derived target) | High | High |
| Zhumagozhayev 2026 (2026) | Some concerns | Some concerns | Some concerns (expression-derived target) | Some concerns | Some concerns |

# Table S6B. Reporting and reproducibility characteristics in supervised studies (n=59)

These items are descriptive reporting/reproducibility characteristics, not risk-of-bias domains.

| **Study** | **Preprocessing detail** | **Validation detail** | **Data provenance** | **Code/software** | **Confidence intervals** |
| --- | --- | --- | --- | --- | --- |
| Tao M 2019 (2019) | Adequate | Adequate | Yes | No/unclear | No/unclear |
| Xu J 2019 (2019) | Adequate | Adequate | Yes | No/unclear | No/unclear |
| Chierici M 2020 (2020) | Adequate | Adequate | Yes | Yes | Yes |
| Lin Y 2020 (2020) | Adequate | Adequate | Yes | Yes | No/unclear |
| Mohaiminul Islam M 2020 (2020) | Adequate | Adequate | Yes | No/unclear | No/unclear |
| Zhang 2020 (2020) | Adequate | Adequate | Yes | No/unclear | No/unclear |
| El-Nabawy 2021 (2021) | Adequate | Partial | Yes | No/unclear | No/unclear |
| Jung I 2021 (2021) | Adequate | Adequate | Yes | Yes | No/unclear |
| Zhang L 2021 (2021) | Partial | Adequate | Yes | Yes (AutoGGN software site) | No |
| Khan D 2022 (2022) | Adequate | Adequate | Yes | Yes | No/unclear |
| Li X 2022 (2022) | Adequate | Adequate | Yes | Yes | No/unclear |
| Meshoul 2022 (2022) | Adequate | Partial | Yes | Yes | No/unclear |
| Benkirane H 2023 (2023) | Adequate | Adequate | Yes | Yes | No/unclear |
| Choi JM 2023 (2023) | Adequate | Adequate | Yes | Yes | No/unclear |
| Guo H 2023 (2023) | Partial | Adequate | Yes | Yes (GCN_multiomics repository) | No |
| Kesimoglu ZN 2023 (2023) | Adequate | Adequate | Yes | Yes | No/unclear |
| Lupat 2023 (2023) | Adequate | Adequate | Yes | Yes | No/unclear |
| Unlu Yazici M 2023 (2023) | Adequate | Adequate | Yes | Yes | No/unclear |
| Cheng L 2024 (2024) | Partial | Partial | Yes | No/unclear | No/unclear |
| Gygi JP 2024 (2024) | Adequate | Adequate | Yes | Yes | Yes |
| Huang Y 2024 (2024) | Adequate | Partial | Yes | Yes | No/unclear |
| Li B 2024 (2024) | Adequate | Partial | Yes | No/unclear | No/unclear |
| Liu Z 2024 (2024) | Adequate | Adequate | Yes | No/unclear | No/unclear |
| Ren Y 2024 (2024) | Adequate | Adequate | Yes | No/unclear | No/unclear |
| Shen J 2024 (2024) | Adequate | Adequate | Partial | Yes | No/unclear |
| Tang X 2024 (2024) | Adequate | Adequate | Yes | Yes | No/unclear |
| Tanvir 2024 (2024) | Partial | Partial | Yes | Yes | No/unclear |
| Wang H 2024 (2024) | Partial | Adequate | Yes | Yes | No |
| Wang J 2024 (2024) | Adequate | Partial | Partial | No/unclear | No/unclear |
| Wu J 2024 (2024) | Adequate | Partial | Yes | Yes | No/unclear |
| Yang S 2024 (2024) | Adequate | Adequate | Yes | No/unclear | No/unclear |
| Yao 2024 (2024) | Adequate | Partial | Partial | Yes | Yes |
| Zeng 2024 (2024) | Adequate | Adequate | Yes | Yes | No/unclear |
| Zhang 2024 (2024) | Adequate | Partial | Yes | No/unclear | No/unclear |
| Baena-Miret 2025 (2025) | Adequate | Partial | Yes | Yes | No/unclear |
| Omran MM 2025 (2025) | Adequate | Adequate | Yes | No/unclear | No/unclear |
| Patel JC 2025 (2025) | Adequate | Adequate | Yes | Yes | No/unclear |
| Tan CY 2025 (2025) | Adequate | Adequate | Yes | Yes | No/unclear |
| Tan K 2025 (2025) | Partial | Adequate | Yes | Yes | No |
| Unlu Yazici M 2025 (2025) | Adequate | Adequate | Yes | Yes | No/unclear |
| Zhang G 2025 (2025) | Adequate | Adequate | Yes | Yes | No |
| Zhao G 2025 (2025) | Partial | Adequate | Yes | No/unclear | No |
| Atfa 2026 (2026) | Adequate | Adequate | Yes | No/unclear | No/unclear |
| Fu X 2026 (2026) | Adequate | Adequate | Yes | No/unclear | Yes |
| Guo M 2026 (2026) | Adequate | Adequate | Yes | Yes | No/unclear |
| Huang Y 2026 (2026) | Partial | Partial | Yes | Yes | No |
| Lan G 2026 (2026) | Partial | Adequate | Yes | No/unclear | No |
| Li C 2026 (2026) | Adequate | Adequate | Yes | No/unclear | Yes |
| Li M 2026 (2026) | Partial | Adequate | Yes | No/unclear | No |
| Li Q 2026 (2026) | Partial | Adequate | Yes | No/unclear | No |
| Meng P 2026 (2026) | Adequate | Adequate | Yes | Yes | No/unclear |
| Mu N 2026 (2026) | Partial | Partial | Yes | Yes | No |
| Siam MBH 2026 (2026) | Adequate | Partial | Partial | No/unclear | Yes |
| Subramaniam M 2026 (2026) | Partial | Partial/internally inconsistent | Yes | No/unclear | Yes (bootstrap) |
| Wu H 2026 (2026) | Adequate | Adequate | Yes | Yes | No |
| Yang Y 2026 (2026) | Adequate | Adequate | Yes | No/unclear | No |
| Zhang G 2026 (2026) | Partial | Adequate | Yes | Yes | No |
| Zhumagozhayev 2026 (2026) | Partial | Adequate | Yes | No/unclear | No/unclear |
| Zhumagozhayev 2026 (2026) | Adequate | Adequate | Yes | No/unclear | Yes |

# Table S6C. Validation and clinical-readiness characteristics in supervised studies (n=59)

Complete external model validation required the trained multi-omics pipeline, rather than only a signature, representation or biological association, to be evaluated in an independent patient population.

| **Study** | **Complete external model** | **Prospective** | **Nested full pipeline** | **Calibration** | **Uncertainty** | **Fold-contained preprocessing** | **Imbalance handled** | **Missing/progressive modalities** |
| --- | --- | --- | --- | --- | --- | --- | --- | --- |
| Tao M 2019 (2019) | No | No | No/unclear | No | No | No/unclear | No/unclear | No |
| Xu J 2019 (2019) | No | No | No/unclear | No | No | No/unclear | No/unclear | No |
| Chierici M 2020 (2020) | No | No | No/unclear | No | No | Yes | No/unclear | No |
| Lin Y 2020 (2020) | No | No | No/unclear | No | No | No/unclear | Yes | No |
| Mohaiminul Islam M 2020 (2020) | No | No | No/unclear | No | No | No/unclear | Yes | No |
| Zhang 2020 (2020) | No | No | No/unclear | No | No | No/unclear | No/unclear | No |
| El-Nabawy 2021 (2021) | No | No | No/unclear | No | No | No/unclear | Yes | No |
| Jung I 2021 (2021) | No | No | No/unclear | No | No | Yes | No/unclear | No |
| Zhang L 2021 (2021) | No | No | No | No | No | No/unclear | No/unclear | No |
| Khan D 2022 (2022) | No | No | No/unclear | No | No | No/unclear | Yes | No |
| Li X 2022 (2022) | No | No | No/unclear | No | No | No/unclear | No/unclear | No |
| Meshoul 2022 (2022) | No | No | No/unclear | No | No | No/unclear | No/unclear | No |
| Benkirane H 2023 (2023) | No | No | No/unclear | No | No | No/unclear | No/unclear | No |
| Choi JM 2023 (2023) | No | No | No/unclear | No | No | Yes | No/unclear | No |
| Guo H 2023 (2023) | No | No | No | No | No | No/unclear | No/unclear | No |
| Kesimoglu ZN 2023 (2023) | No | No | No/unclear | No | No | No/unclear | No/unclear | No |
| Lupat 2023 (2023) | Yes (METABRIC to TCGA) | No | No/unclear | No | No | No/unclear | No/unclear | No |
| Unlu Yazici M 2023 (2023) | No | No | No/unclear | No | No | No/unclear | No/unclear | No |
| Cheng L 2024 (2024) | No | No | No/unclear | No | No | No/unclear | No/unclear | No |
| Gygi JP 2024 (2024) | No | No | No/unclear | No | No | No/unclear | No/unclear | No |
| Huang Y 2024 (2024) | No | No | No/unclear | No | No | No/unclear | No/unclear | No |
| Li B 2024 (2024) | No | No | No/unclear | No | No | No/unclear | No/unclear | No |
| Liu Z 2024 (2024) | No | No | No/unclear | No | No | No/unclear | No/unclear | No |
| Ren Y 2024 (2024) | No | No | No/unclear | No | No | No/unclear | No/unclear | No |
| Shen J 2024 (2024) | No | No | No/unclear | No | No | No/unclear | No/unclear | No |
| Tang X 2024 (2024) | No | No | No/unclear | No | No | No/unclear | No/unclear | No |
| Tanvir 2024 (2024) | No | No | No/unclear | No | No | No/unclear | No/unclear | No |
| Wang H 2024 (2024) | No | No | No | No | Yes | No/unclear | No/unclear | No |
| Wang J 2024 (2024) | No | No | No/unclear | No | No | No/unclear | No/unclear | No |
| Wu J 2024 (2024) | No | No | No/unclear | No | No | No/unclear | No/unclear | No |
| Yang S 2024 (2024) | No | No | No/unclear | No | No | No/unclear | No/unclear | No |
| Yao 2024 (2024) | No | No | No/unclear | No | No | No/unclear | No/unclear | No |
| Zeng 2024 (2024) | No | No | No/unclear | No | No | No/unclear | No/unclear | No |
| Zhang 2024 (2024) | No | No | No/unclear | No | No | No/unclear | Yes | No |
| Baena-Miret 2025 (2025) | No | No | No/unclear | No | No | No/unclear | No/unclear | Yes |
| Omran MM 2025 (2025) | No | No | No/unclear | No | No | No/unclear | No/unclear | No |
| Patel JC 2025 (2025) | No | No | No/unclear | No | No | No/unclear | Yes | No |
| Tan CY 2025 (2025) | No | No | No/unclear | No | No | Yes | No/unclear | No |
| Tan K 2025 (2025) | No | No | No | No | No | No/unclear | No/unclear | No |
| Unlu Yazici M 2025 (2025) | No | No | No/unclear | No | No | No/unclear | Yes | No |
| Zhang G 2025 (2025) | No | No | No | No | No | Yes | Yes (inverse-frequency loss) | No |
| Zhao G 2025 (2025) | No | No | No | No | No | No/unclear | Yes (undersampling ensemble) | No |
| Atfa 2026 (2026) | No | No | No/unclear | No | No | Yes | Yes | No |
| Fu X 2026 (2026) | No | No | No/unclear | No | No | Yes | No/unclear | No |
| Guo M 2026 (2026) | No | No | No/unclear | No | No | No/unclear | No/unclear | No |
| Huang Y 2026 (2026) | No | No | No | No | Yes | No/unclear | No/unclear | Yes |
| Lan G 2026 (2026) | No | No | No | No | No | No/unclear | No/unclear | No |
| Li C 2026 (2026) | No | No | No/unclear | Yes | No | Yes | No/unclear | No |
| Li M 2026 (2026) | No | No | No | No | No | No/unclear | No/unclear | No |
| Li Q 2026 (2026) | No | No | No | No | No | No/unclear | No/unclear | No |
| Meng P 2026 (2026) | No | No | No/unclear | No | No | No/unclear | No/unclear | No |
| Mu N 2026 (2026) | No | No | No | No | Yes | No/unclear | No/unclear | Yes |
| Siam MBH 2026 (2026) | No | No | No/unclear | No | No | No/unclear | No/unclear | No |
| Subramaniam M 2026 (2026) | No | No | No | No | Yes (credal intervals) | No/unclear | No/unclear | No |
| Wu H 2026 (2026) | No | No | No | No | No | Yes | No/unclear | No |
| Yang Y 2026 (2026) | No | No | No | No | No | Yes | Yes (balanced class weights) | No |
| Zhang G 2026 (2026) | No | No | No | No | No | No/unclear | No/unclear | No |
| Zhumagozhayev 2026 (2026) | No | No | No/unclear | No | No | No/unclear | No/unclear | No |
| Zhumagozhayev 2026 (2026) | No | No | No/unclear | No | No | Yes | Yes | No |

# Table S6D. Interpretability characteristics in supervised studies (n=59)

An explanation was counted as evaluated only when stability, fidelity or robustness was explicitly assessed. Enrichment or plausibility checking was kept separate from external replication.

| **Study** | **Explicit XAI/feature attribution** | **Explanation evaluated** | **Biological plausibility/enrichment** | **External biological replication** |
| --- | --- | --- | --- | --- |
| Tao M 2019 (2019) | None reported | No/unclear | No/unclear | No |
| Xu J 2019 (2019) | None reported | No/unclear | No/unclear | No |
| Chierici M 2020 (2020) | None reported | No/unclear | Yes | No |
| Lin Y 2020 (2020) | None reported | No/unclear | No/unclear | No |
| Mohaiminul Islam M 2020 (2020) | None reported | No/unclear | No/unclear | No |
| Zhang 2020 (2020) | None reported | No/unclear | No/unclear | No |
| El-Nabawy 2021 (2021) | None reported | No/unclear | No/unclear | No |
| Jung I 2021 (2021) | None reported | No/unclear | No/unclear | No |
| Zhang L 2021 (2021) | SHAP | No | Yes | No |
| Khan D 2022 (2022) | None reported | No/unclear | No/unclear | No |
| Li X 2022 (2022) | None reported | No/unclear | Yes | No |
| Meshoul 2022 (2022) | SHAP | No/unclear | No/unclear | No |
| Benkirane H 2023 (2023) | SHAP | No/unclear | Yes | No |
| Choi JM 2023 (2023) | None reported | No/unclear | No/unclear | No |
| Guo H 2023 (2023) | Layer-wise relevance propagation | No | Yes | No |
| Kesimoglu ZN 2023 (2023) | None reported | No/unclear | No/unclear | No |
| Lupat 2023 (2023) | None reported | No/unclear | No/unclear | No |
| Unlu Yazici M 2023 (2023) | None reported | No/unclear | Yes | No |
| Cheng L 2024 (2024) | None reported | No/unclear | Yes | No |
| Gygi JP 2024 (2024) | None reported | No/unclear | No/unclear | No |
| Huang Y 2024 (2024) | None reported | No/unclear | No/unclear | No |
| Li B 2024 (2024) | None reported | No/unclear | No/unclear | No |
| Liu Z 2024 (2024) | None reported | Yes | No/unclear | No |
| Ren Y 2024 (2024) | None reported | No/unclear | No/unclear | No |
| Shen J 2024 (2024) | None reported | No/unclear | No/unclear | No |
| Tang X 2024 (2024) | SHAP | No/unclear | Yes | No |
| Tanvir 2024 (2024) | None reported | No/unclear | No/unclear | No |
| Wang H 2024 (2024) | Feature-importance/ablation ranking | No | Yes | No |
| Wang J 2024 (2024) | None reported | No/unclear | No/unclear | No |
| Wu J 2024 (2024) | None reported | No/unclear | Yes | No |
| Yang S 2024 (2024) | None reported | No/unclear | Yes | No |
| Yao 2024 (2024) | None reported | No/unclear | No/unclear | No |
| Zeng 2024 (2024) | None reported | No/unclear | No/unclear | No |
| Zhang 2024 (2024) | None reported | No/unclear | No/unclear | No |
| Baena-Miret 2025 (2025) | None reported | No/unclear | No/unclear | No |
| Omran MM 2025 (2025) | SHAP | No/unclear | Yes | No |
| Patel JC 2025 (2025) | SHAP | No/unclear | No/unclear | No |
| Tan CY 2025 (2025) | Integrated Gradients | No/unclear | Yes | Yes |
| Tan K 2025 (2025) | None reported | No | Yes | No |
| Unlu Yazici M 2025 (2025) | None reported | No/unclear | Yes | No |
| Zhang G 2025 (2025) | Graph feature-importance scores | No | Yes | No |
| Zhao G 2025 (2025) | None reported | No | No/unclear | No |
| Atfa 2026 (2026) | SHAP | No/unclear | Yes | No |
| Fu X 2026 (2026) | SHAP | No/unclear | Yes | No |
| Guo M 2026 (2026) | GNNExplainer | No/unclear | No/unclear | No |
| Huang Y 2026 (2026) | None reported | No | No/unclear | No |
| Lan G 2026 (2026) | None reported | No | No/unclear | No |
| Li C 2026 (2026) | Integrated Gradients | No/unclear | No/unclear | No |
| Li M 2026 (2026) | Masking-based feature importance | No | Yes | No |
| Li Q 2026 (2026) | Guided Grad-CAM | No | Yes | Yes |
| Meng P 2026 (2026) | SHAP | No/unclear | Yes | No |
| Mu N 2026 (2026) | None reported | No | No/unclear | No |
| Siam MBH 2026 (2026) | SHAP; GNNExplainer | No/unclear | No/unclear | No |
| Subramaniam M 2026 (2026) | None reported | No | No/unclear | No |
| Wu H 2026 (2026) | Attention/feature-importance scores | No | Yes | No |
| Yang Y 2026 (2026) | Permutation feature importance | No | Yes | No |
| Zhang G 2026 (2026) | Attention-derived feature importance | No | Yes | No |
| Zhumagozhayev 2026 (2026) | LRP | No/unclear | Yes | No |
| Zhumagozhayev 2026 (2026) | None reported | No/unclear | No/unclear | No |

# Table S6E. Validity characteristics in subtype-discovery studies (n=11)

Discovery studies were assessed with endpoint-relevant characteristics rather than the supervised prediction risk-of-bias domains.

| **Study** | **DOI** | **Resampling/perturbation stability** | **Analytic sensitivity** | **Biological/clinical association** | **Independent recovery** | **Code** |
| --- | --- | --- | --- | --- | --- | --- |
| Qi L 2021 (2021) | [10.3389/fgene.2021.607817](https://doi.org/10.3389/fgene.2021.607817) | No/unclear | No/unclear | No/unclear | Yes | No/unclear |
| Yang H 2021 (2021) | [10.1093/bioinformatics/btab109](https://doi.org/10.1093/bioinformatics/btab109) | Yes | Yes | Yes | No | Yes |
| Pouryahya M 2022 (2022) | [10.1109/tcbb.2020.3039511](https://doi.org/10.1109/tcbb.2020.3039511) | No/unclear | No/unclear | Yes | No | Yes |
| Valle F 2022 (2022) | [10.3390/cancers14051150](https://doi.org/10.3390/cancers14051150) | No/unclear | No/unclear | No/unclear | No | Yes |
| Viaud G 2022 (2022) | [10.1109/tcbb.2021.3060340](https://doi.org/10.1109/tcbb.2021.3060340) | No/unclear | Yes | No/unclear | No | No/unclear |
| Bong F 2025 (2025) | [10.1093/nar/gkaf844](https://doi.org/10.1093/nar/gkaf844) | No/unclear | No/unclear | Yes | Yes | Yes |
| Hu GM 2025 (2025) | [10.1093/bib/bbaf452](https://doi.org/10.1093/bib/bbaf452) | Yes | Yes | Yes | No | No/unclear |
| Muneeba Afzal Mukhdoomi 2025 (2025) | [10.12732/ijam.v38i2s.71](https://doi.org/10.12732/ijam.v38i2s.71) | No/unclear | No/unclear | Yes | No | No/unclear |
| Pateras J 2025 (2025) | [10.3390/ijms26041707](https://doi.org/10.3390/ijms26041707) | No/unclear | No/unclear | Yes | No | No/unclear |
| Wang J 2026 (2026) | [10.1007/s12539-025-00789-1](https://doi.org/10.1007/s12539-025-00789-1) | Yes | Yes | Yes | No | Yes |
| Wang T 2026 (2026) | [10.1016/j.intonc.2025.12.001](https://doi.org/10.1016/j.intonc.2025.12.001) | Yes | Yes | Yes | Yes | Yes |

# Table S7. PAM50 target-dependence audit (n=57)

Incremental gain records an author-reported improvement in at least one prespecified metric under a matched within-study comparison. It does not by itself establish statistical significance, clinical benefit or external transportability.

| **Study** | **DOI** | **Expression input** | **Label origin clear** | **PAM50 genes/proxies excluded** | **Matched expression-only baseline** | **Incremental non-expression gain** | **Complete external multi-omics model** |
| --- | --- | --- | --- | --- | --- | --- | --- |
| Tao M 2019 (2019) | [10.3390/genes10030200](https://doi.org/10.3390/genes10030200) | Yes | No/unclear | No/unclear | Yes | Yes | No |
| Xu J 2019 (2019) | [10.1186/s12859-019-3116-7](https://doi.org/10.1186/s12859-019-3116-7) | Yes | No/unclear | No/unclear | Yes | Yes | No |
| Chierici M 2020 (2020) | [10.3389/fonc.2020.01065](https://doi.org/10.3389/fonc.2020.01065) | Yes | No/unclear | No/unclear | No/unclear | No/unclear | No |
| Lin Y 2020 (2020) | [10.3390/genes11080888](https://doi.org/10.3390/genes11080888) | Yes | No/unclear | No/unclear | Yes | Yes | No |
| Mohaiminul Islam M 2020 (2020) | [10.1016/j.csbj.2020.08.005](https://doi.org/10.1016/j.csbj.2020.08.005) | Yes | Yes | No/unclear | Yes | Yes | No |
| Zhang 2020 (2020) | [10.1109/access.2019.2960373](https://doi.org/10.1109/access.2019.2960373) | Yes | Yes | No/unclear | No/unclear | No/unclear | No |
| El-Nabawy 2021 (2021) | [10.3390/math9131574](https://doi.org/10.3390/math9131574) | Yes | Yes | No/unclear | Yes | No | No |
| Jung I 2021 (2021) | [10.3389/fgene.2021.682841](https://doi.org/10.3389/fgene.2021.682841) | Yes | Yes | No/unclear | Yes | Yes | No |
| Zhang L 2021 (2021) | [10.1016/j.ailsci.2021.100019](https://doi.org/10.1016/j.ailsci.2021.100019) | Yes | Yes | No/unclear | Yes | Yes | No |
| Khan D 2022 (2022) | [10.3390/jpm12050674](https://doi.org/10.3390/jpm12050674) | Yes | Yes | No/unclear | Yes | Yes | No |
| Li X 2022 (2022) | [10.3389/fgene.2022.806842](https://doi.org/10.3389/fgene.2022.806842) | Yes | Yes | No/unclear | No/unclear | No/unclear | No |
| Meshoul 2022 (2022) | [10.3390/math10224271](https://doi.org/10.3390/math10224271) | Yes | No/unclear | No/unclear | Yes | Yes | No |
| Benkirane H 2023 (2023) | [10.1371/journal.pcbi.1010921](https://doi.org/10.1371/journal.pcbi.1010921) | Yes | Yes | No/unclear | No/unclear | No/unclear | No |
| Choi JM 2023 (2023) | [10.1186/s12859-023-05273-5](https://doi.org/10.1186/s12859-023-05273-5) | Yes | Yes | No/unclear | Yes | Yes | No |
| Guo H 2023 (2023) | [10.1093/bfgp/elad013](https://doi.org/10.1093/bfgp/elad013) | Yes | Yes | No/unclear | Yes | Yes | No |
| Kesimoglu ZN 2023 (2023) | [10.1093/nargab/lqad063](https://doi.org/10.1093/nargab/lqad063) | Yes | Yes | No/unclear | Yes | Yes | No |
| Lupat 2023 (2023) | [10.1109/access.2023.3240515](https://doi.org/10.1109/access.2023.3240515) | Yes | Yes | No/unclear | No/unclear | No/unclear | Yes |
| Unlu Yazici M 2023 (2023) | [10.3389/fgene.2023.1093326](https://doi.org/10.3389/fgene.2023.1093326) | Yes | Yes | No/unclear | Yes | Yes | No |
| Cheng L 2024 (2024) | [10.1186/s12859-024-05989-y](https://doi.org/10.1186/s12859-024-05989-y) | Yes | Yes | No/unclear | Yes | Yes | No |
| Gygi JP 2024 (2024) | [10.1093/bioinformatics/btae202](https://doi.org/10.1093/bioinformatics/btae202) | Yes | Yes | No/unclear | No/unclear | No/unclear | No |
| Huang Y 2024 (2024) | [10.1186/s12859-024-05749-y](https://doi.org/10.1186/s12859-024-05749-y) | Yes | No/unclear | No/unclear | Yes | Yes | No |
| Li B 2024 (2024) | [10.1186/s12859-023-05622-4](https://doi.org/10.1186/s12859-023-05622-4) | Yes | No/unclear | No/unclear | Yes | Yes | No |
| Liu Z 2024 (2024) | [10.3389/fgene.2024.1488683](https://doi.org/10.3389/fgene.2024.1488683) | Yes | No/unclear | No/unclear | Yes | No | No |
| Ren Y 2024 (2024) | [10.3389/fgene.2024.1363896](https://doi.org/10.3389/fgene.2024.1363896) | Yes | No/unclear | No/unclear | Yes | Yes | No |
| Shen J 2024 (2024) | [10.3389/fbinf.2024.1403826](https://doi.org/10.3389/fbinf.2024.1403826) | Yes | No/unclear | No/unclear | Yes | Yes | No |
| Tang X 2024 (2024) | [10.1093/nar/gkae915](https://doi.org/10.1093/nar/gkae915) | Yes | Yes | No/unclear | Yes | Yes | No |
| Tanvir 2024 (2024) | [10.3390/ijms25052788](https://doi.org/10.3390/ijms25052788) | Yes | No/unclear | No/unclear | No/unclear | No/unclear | No |
| Wang H 2024 (2024) | [10.1093/bioinformatics/btae159](https://doi.org/10.1093/bioinformatics/btae159) | Yes | Yes | No/unclear | No/unclear | No/unclear | No |
| Wang J 2024 (2024) | [10.1186/s12864-024-09985-7](https://doi.org/10.1186/s12864-024-09985-7) | Yes | Yes | No/unclear | Yes | Yes | No |
| Wu J 2024 (2024) | [10.1186/s12864-024-11112-5](https://doi.org/10.1186/s12864-024-11112-5) | Yes | Yes | No/unclear | Yes | Yes | No |
| Yao 2024 (2024) | [10.1186/s13040-024-00360-6](https://doi.org/10.1186/s13040-024-00360-6) | Yes | Yes | No/unclear | No/unclear | No/unclear | No |
| Zeng 2024 (2024) | [10.3390/app14072728](https://doi.org/10.3390/app14072728) | Yes | No/unclear | No/unclear | No/unclear | No/unclear | No |
| Zhang 2024 (2024) | [10.62617/mcb785](https://doi.org/10.62617/mcb785) | Yes | Yes | No/unclear | Yes | Yes | No |
| Baena-Miret 2025 (2025) | [10.3390/app15073650](https://doi.org/10.3390/app15073650) | Yes | No/unclear | No/unclear | No/unclear | No/unclear | No |
| Omran MM 2025 (2025) | [10.1186/s12967-025-06662-5](https://doi.org/10.1186/s12967-025-06662-5) | Yes | Yes | No/unclear | Yes | Yes | No |
| Patel JC 2025 (2025) | [10.1093/bioadv/vbaf116](https://doi.org/10.1093/bioadv/vbaf116) | Yes | Yes | No/unclear | Yes | Yes | No |
| Tan CY 2025 (2025) | [10.1186/s12859-025-06111-6](https://doi.org/10.1186/s12859-025-06111-6) | Yes | No/unclear | No/unclear | Yes | Yes | No |
| Tan K 2025 (2025) | [10.1109/jbhi.2025.3624554](https://doi.org/10.1109/jbhi.2025.3624554) | Yes | Yes | No/unclear | Yes | Yes | No |
| Zhang G 2025 (2025) | [10.1039/d5mo00221d](https://doi.org/10.1039/d5mo00221d) | Yes | Yes | No/unclear | Yes | Yes | No |
| Zhao G 2025 (2025) | [10.1016/j.rinam.2025.100605](https://doi.org/10.1016/j.rinam.2025.100605) | Yes | No/unclear | No/unclear | No/unclear | No/unclear | No |
| Atfa 2026 (2026) | [10.14569/ijacsa.2026.0170537](https://doi.org/10.14569/ijacsa.2026.0170537) | Yes | Yes | No/unclear | Yes | Yes | No |
| Fu X 2026 (2026) | [10.3390/genes17080865](https://doi.org/10.3390/genes17080865) | Yes | Yes | No/unclear | No/unclear | No/unclear | No |
| Guo M 2026 (2026) | [10.1186/s12859-026-06404-4](https://doi.org/10.1186/s12859-026-06404-4) | Yes | No/unclear | No/unclear | No/unclear | No/unclear | No |
| Huang Y 2026 (2026) | [10.1016/j.compbiomed.2026.111860](https://doi.org/10.1016/j.compbiomed.2026.111860) | Yes | No/unclear | No/unclear | Yes | No | No |
| Lan G 2026 (2026) | [10.1155/ijcp/6988098](https://doi.org/10.1155/ijcp/6988098) | Yes | Yes | No/unclear | Yes | Yes | No |
| Li C 2026 (2026) | [10.3389/fmed.2026.1790437](https://doi.org/10.3389/fmed.2026.1790437) | Yes | Yes | No/unclear | Yes | Yes | No |
| Li M 2026 (2026) | [10.1016/j.compbiolchem.2025.108665](https://doi.org/10.1016/j.compbiolchem.2025.108665) | Yes | Yes | No/unclear | No/unclear | No/unclear | No |
| Li Q 2026 (2026) | [10.1109/jbhi.2025.3595381](https://doi.org/10.1109/jbhi.2025.3595381) | Yes | No/unclear | No/unclear | Yes | Yes | No/unclear (locked cross-cohort transfer not demonstrated) |
| Meng P 2026 (2026) | [10.3390/ijms27104237](https://doi.org/10.3390/ijms27104237) | Yes | No/unclear | No/unclear | Yes | Yes | No |
| Mu N 2026 (2026) | [10.1016/j.cmpb.2025.109179](https://doi.org/10.1016/j.cmpb.2025.109179) | Yes | No/unclear | No/unclear | Yes | No | No |
| Siam MBH 2026 (2026) | [10.1371/journal.pone.0344754](https://doi.org/10.1371/journal.pone.0344754) | Yes | Yes | No/unclear | No/unclear | No/unclear | No |
| Subramaniam M 2026 (2026) | [10.1016/j.bspc.2025.109298](https://doi.org/10.1016/j.bspc.2025.109298) | Yes | No/unclear | No/unclear | No/unclear | No/unclear | No |
| Wu H 2026 (2026) | [10.1007/s12539-026-00828-5](https://doi.org/10.1007/s12539-026-00828-5) | Yes | Yes | No/unclear | Yes | Yes | No |
| Yang Y 2026 (2026) | [10.1080/10255842.2026.2642810](https://doi.org/10.1080/10255842.2026.2642810) | Yes | Yes | No/unclear | No/unclear | No/unclear | No |
| Zhang G 2026 (2026) | [10.1016/j.cmpb.2026.109562](https://doi.org/10.1016/j.cmpb.2026.109562) | Yes | Yes | No/unclear | Yes | Yes | No |
| Zhumagozhayev 2026 (2026) | [10.1109/access.2026.3695279](https://doi.org/10.1109/access.2026.3695279) | Yes | Yes | No/unclear | Yes | Yes | No |
| Zhumagozhayev 2026 (2026) | [10.23950/jcmk/18568](https://doi.org/10.23950/jcmk/18568) | Yes | Yes | No/unclear | Yes | Yes | No |

# Table S8. Underlying patient-population map

Publication counts overlap because one publication can use more than one population. Reprocessed, resampled or differently filtered versions of the same named resource were not treated as independent populations.

| **Patient population** | **Role** | **Included publications** | **Independence rule** |
| --- | --- | --- | --- |
| TCGA-BRCA | Development/internal validation; occasional external test | 62 | All TCGA-BRCA subsets and reprocessed benchmark files count as one underlying patient population. |
| METABRIC | Development, internal validation or transfer | 9 | Repeated use of METABRIC does not create independent populations. |
| GSE22220 | External subtype recovery/transfer | 1 | Independent named patient series. |
| AURORA-US | External biological/subtype replication | 1 | Independent named patient series. |
| GSE84207 | External methylation/biological replication | 1 | Independent named patient series; not a complete multi-omics model test. |
| GSE202203 | External expression-based subtype recovery | 1 | Independent named patient series. |
| GSE96058 | External expression-based subtype recovery | 1 | Independent named patient series. |
| Netherlands Cancer Institute (NKI) | External expression-based subtype recovery | 1 | Independent named patient series. |
